# BM-MSC-derived migrasomes reverse stroke-induced thymic atrophy and immunosuppression via Pin1 delivery to thymic epithelial cells

**DOI:** 10.1186/s12974-025-03604-2

**Published:** 2025-11-15

**Authors:** Haotong Yi, Mengyan Hu, Liling Yuan, Xiaotao Su, Shilin Wu, Tiemei Li, Shisi Wang, Xinmei Kang, Yuxin Liu, Zhiruo Liu, Qin Qin, Weihua Yu, Yifan Li, Wei Qiu, Wei Cai, Zhengqi Lu

**Affiliations:** 1 Department of Neurology, Mental and Neurological Disease Research Center, The Third Affiliated Hospital of Sun Yat-sen University, 600 Tianhe Road, Guangzhou, Guangdong Province 510630 China; 2 Guangdong Provincial Key Laboratory of Brain Function and Disease, Guangzhou, Guangdong Province China; 3 Guangzhou Saijun Biological Technology Co. Ltd, Guangzhou, Guangdong Province China

**Keywords:** Migrasomes, BM-MSC, Thymus, Stroke, Thymic epithelial cells, Immunosuppression

## Abstract

Acute ischemic stroke triggers immunosuppression, yet existing therapies struggle to balance neuroprotection with poststroke immunosuppression. We demonstrated that bone marrow mesenchymal stem cells (BM-MSC) reverse stroke-induced thymic atrophy by promoting T-cell differentiation and restoring peripheral T-cell populations. Bulk RNA sequencing of BM-MSC-treated thymuses revealed enhanced proliferative signatures. Mechanistically, BM-MSC secrete migrasomes (organelles derived from migrating cells) that traverse the blood‒thymus barrier. Single-cell RNA sequencing analysis demonstrated that migrasome-mediated proliferation occurred specifically in medullary thymic epithelial cell I (mTECI) subpopulations. Proteomic profiling via liquid chromatography‒tandem mass spectrometry (LC‒MS/MS) identified Pin1—a cell cycle regulator—as the predominant cargo in BM-MSC-derived migrasomes. In vivo and in vitro studies confirmed migrasome-mediated thymic epithelial proliferation, T-cell niche reconstruction, and immune homeostasis restoration. Migrasome monotherapy improved neurological deficits and survival rates in stroke model mice, demonstrating dual neuroprotective-immunomodulatory efficacy. This work addresses the clinical dilemma between neuroprotection and immunosuppression alleviation, establishing migrasomes as a cell-free therapeutic strategy for poststroke immunotherapy.

**Supplementary Information:**

The online version contains supplementary material available at 10.1186/s12974-025-03604-2.

## Introduction

Following acute ischemic stroke (AIS), activation of the sympathetic nervous system (SNS) and the hypothalamic‒pituitary‒adrenal (HPA) axis induces an immunosuppressive state to mitigate neuroinflammatory damage caused by blood‒brain barrier disruption [1–3]. However, this compensatory response elevates immunosuppressive factors (e.g., IL-10 and glucocorticoids), leading to lymphocytopenia and poststroke immunosuppression (PICS), which heightens susceptibility to stroke-associated infections (SAIs) and exacerbates neurological deficits and mortality [4, 5]. Current therapies targeting SNS/HPA axis inhibition or immunosuppressive factor antagonism remain suboptimal because of their interference with endogenous neuroprotective pathways [6–8].

Bone marrow mesenchymal stem cells (BM-MSC) exhibit well-documented immunomodulatory potential, reducing post-AIS neuroinflammation, infarct volume, and SAIs incidence through paracrine mechanisms [9, 10]. For example, BM-MSC enhance pulmonary macrophage phagocytosis to mitigate pneumonia [11] and prevent caspase-1-dependent marginal zone B-cell death in the spleen [12]. Nevertheless, the thymus—the central organ for T-cell development—suffers severe atrophy poststroke due to SNS/HPA axis activation, diminishing T-cell output and aggravating peripheral immunosuppression [13, 14]. The integrity of the thymic microenvironment relies on thymic epithelial cells (TEC) and the blood-thymus barrier (BTB) [15]; however, no studies have explored whether BM-MSC can repair stroke-induced thymic damage.

This gap highlights a critical dilemma in poststroke immunotherapy: current therapies targeting the SNS/HPA axis risk disrupting endogenous neuroprotection, whereas thymic regeneration—a cornerstone of immune recovery—remains unaddressed. We hypothesized that BM-MSC could restore poststroke thymic atrophy and reconstruct T-cell developmental niches to alleviate peripheral immunosuppression.

This study demonstrated that BM-MSC significantly reverse stroke-induced thymic atrophy by promoting medullary thymic epithelial cell (mTEC) proliferation. Mechanistically, while BM-MSC themselves cannot traverse the BTB, their secreted migrasomes—organelles derived from migrating cells [16]—penetrate the BTB to deliver functional cargo to the thymic medulla. Single-cell RNA sequencing (scRNA-seq) analysis revealed that migrasomes primarily drive proliferation in the mTECI subpopulation and restore global thymic epithelial homeostasis. Crucially, BM-MSC treated with blebbistatin (Bleb) [17, 18], or Tspan4-knockdown (*Tspan4*^KD^), in which migrasome production is suppressed, completely abrogate BM-MSC-induced thymic repair. Through liquid chromatography‒tandem mass spectrometry (LC‒MS/MS), we identified Pin1 (peptidyl-prolyl cis-trans isomerase NIMA-interacting 1), a key cell cycle regulator [19], as a prominent cargo protein in BM-MSC-derived migrasomes. Both in vivo and in vitro experiments confirmed that migrasomes deliver Pin1 to mTEC, increasing their proliferation.

This is the first study to demonstrate that BM-MSC utilize migrasomes to bypass the BTB and deliver therapeutic molecules to the thymus, overcoming the limitations of conventional cell-based therapies in targeting central immune organs. Migrasome monotherapy recapitulates the dual benefits of BM-MSC (neuroprotection and immune restoration) while avoiding the ethical and safety concerns associated with stem cell transplantation. These findings not only reveal a novel mechanism by which BM-MSC regulate central immune organs but also pioneer a cell-free strategy for poststroke immunotherapy, resolving the clinical dilemma between neuroprotection and immunosuppression alleviation.

## Results

### Bone marrow mesenchymal stem cells reverse poststroke thymic atrophy and maintain central immune homeostasis

**Fig. 1:**
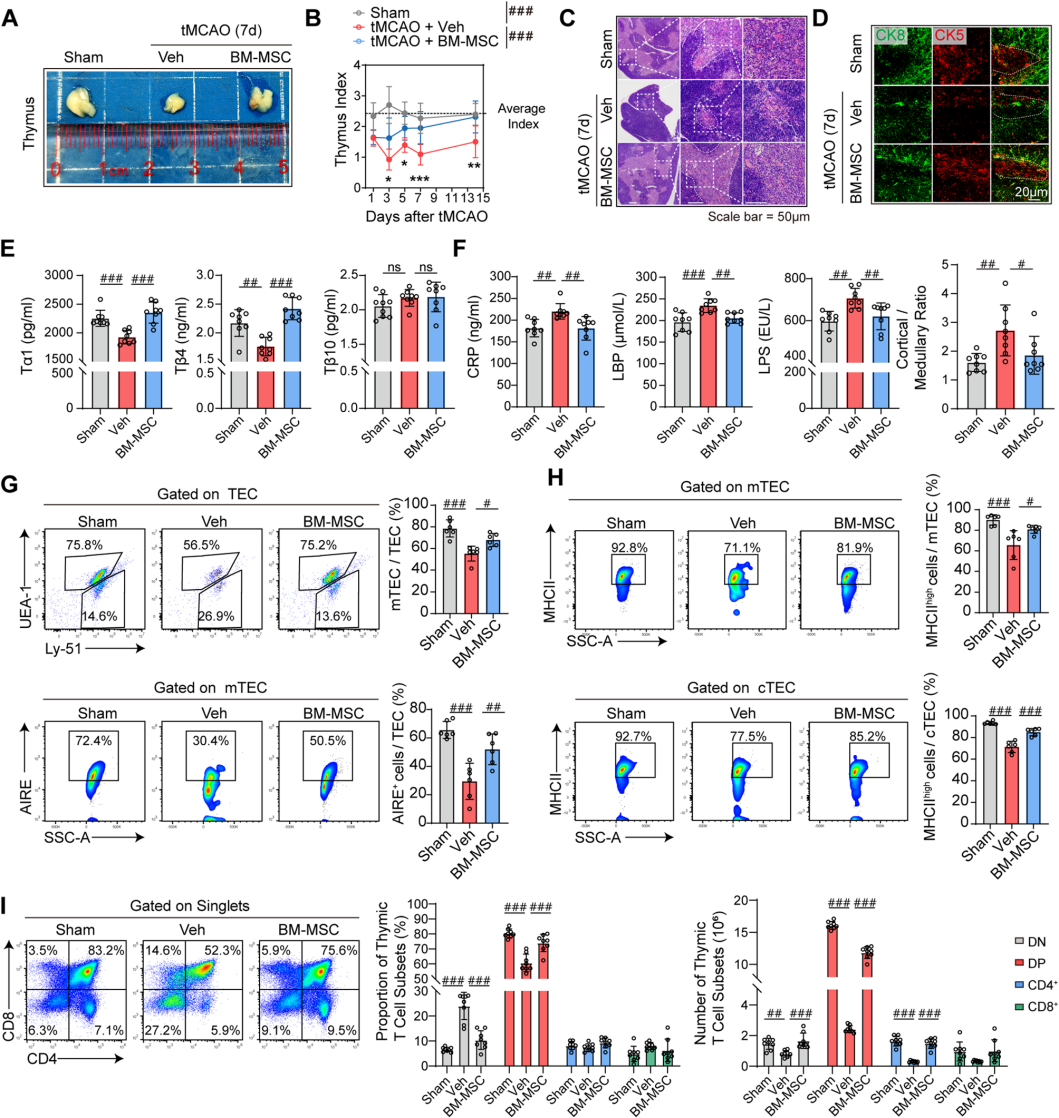
Bone marrow mesenchymal stem cell (BM-MSC) transplantation reverses poststroke thymic atrophy and increases t-cell generation. Male wild-type C57/Bl6 mice underwent 60-minute transient middle cerebral artery occlusion (tMCAO) followed by reperfusion. BM-MSC (2 × 10⁶ cells/mouse) or vehicle (Veh) were administered intravenously as a single dose 2 h postreperfusion. The mice were sacrificed at 1, 3, 5, 7, and 14 days post-tMCAO. **A** Gross thymus images from the Sham, Veh, and BM-MSC groups on day 7 (*n* = 3 per group). **B** Dynamic changes in thymus weight over 14 days (*n* = 10 per group). FDR-corrected *p*-values (*q-values)* were calculated, #*q* < 0.05, ##*q* < 0.01, ###*q* < 0.001 (BM-MSC vs. Veh), one-way ANOVA (mean ± SD). FDR-corrected *p*-values (*q-values)* were calculated, ***q < 0.001 (main effect of group), two-way ANOVA (mean ± SD). **C** Representative hematoxylin and eosin (H&E)-stained thymic sections (*n* = 3 per group). **D** Immunofluorescence staining of the thymus: the medullary marker CK5 (red) and the cortical marker CK8 (green) are shown. The bar graphs below show the cortical/medullary ratios (*n* = 8 per group). FDR-corrected *p*-values (*q-values)* were calculated, #*q* < 0.05, ##*q* < 0.01, by one-way *ANOVA* (mean ± SD). **E** Plasma levels of thymosin α1, β4, and β10 were measured via ELISA (*n* = 8 per group). FDR-corrected *p*-values (*q-values)* were calculated, ##*q* < 0.01, ###*q* < 0.001, by one-way *ANOVA* (mean ± SD). **F** Plasma CRP, LBP, and LPS levels were measured via ELISA (*n* = 8 per group). FDR-corrected *p*-values (*q-values)* were calculated, ##*q* < 0.01, ###*q* < 0.001, by one-way *ANOVA* (mean ± SD). **G** Flow cytometry analysis of thymic epithelial cell (TEC, EPCAM^+^CD45^−^) proportions (*n* = 6 per group). FDR-corrected *p*-values (*q-values)* were calculated, #*q* < 0.05, ###*q* < 0.001, one-way ANOVA (mean ± SD). **H** Flow cytometry analysis of MHC II^+^ and AIRE^+^ mTEC (EPCAM^+^CD45^−^UEA-1^+^Ly51^−^) and MHC II^+^ cTEC (EPCAM^+^CD45^−^UEA^−^Ly51^+^) (*n* = 6 per group). FDR-corrected *p*-values (*q-values)* were calculated, #*q* < 0.05, ##*q* < 0.01, ###*q* < 0.001, one-way ANOVA (mean ± SD). **I** Thymic T-cell subset profiling: proportions and counts of double-negative (DN, CD4^−^CD8^−^), double-positive (DP, CD4^+^CD8^+^), CD4^+^, and CD8^+^ T cells (*n* = 8 per group). FDR-corrected *p*-values (*q-values)* were calculated, ###*q* < 0.001 (BM-MSC vs. Veh), one-way ANOVA (mean ± SD)

Human bone marrow-derived mesenchymal stem cells (BM-MSC) were isolated and validated for their surface marker profiles and adipogenic/osteogenic differentiation capacities (Fig. S1A-B). To evaluate the therapeutic effects of BM-MSC on poststroke thymic atrophy, 8-week-old male wild-type C57BL/6 mice were subjected to 60-minute transient middle cerebral artery occlusion (tMCAO; Fig. S1C), followed by intravenous administration of BM-MSC (2 × 10⁶ cells per mouse) or vehicle (Veh) via the angular vein. Compared with Veh treatment, BM-MSC treatment reversed poststroke thymic atrophy (Fig. 1A), with the thymus index (thymus wet weight (mg)/mouse body weight (g)) recovering more rapidly to baseline levels (Fig. 1B). Consistent with previous findings [10, 11], BM-MSC improved poststroke survival (Fig. S1D), enhanced neurological (Fig. S1E), reduced cerebral infarct volume (Fig. S1F), preserved white matter integrity (MBP staining, Fig. S1G), and mitigated inflammatory cell infiltration (Fig. S2). Hematoxylin‒eosin (H&E) staining revealed a reconstructed cortical-medullary architecture in BM-MSC-treated mice, whereas the Veh group exhibited extensive structural disruption (Fig. 1C). Immunofluorescence analysis revealed that BM-MSC restored the ratio of cortical (CK8^+^) to medullary (CK5^+^) epithelial cells to physiological levels (Fig. 1D). The thymus functions as an endocrine organ capable of secreting bioactive peptides, including thymosin α1 (Tα1), β4 (Tβ4), and β10 (Tβ10) [20]. Existing evidence indicates that Tα1 plays crucial roles in modulating, enhancing, and restoring immune functions [21, 22]. Tβ4 facilitates T-cell differentiation by inducing cytoskeletal reorganization in thymic epithelial cells (TEC) [23], while Tβ10 expression has been associated with tumorigenesis [24]. Compared with the Veh control group, the BM-MSC-treated group presented significantly elevated plasma levels of Tα1 and Tβ4, with no significant effect on the Tβ10 concentration (Fig. 1E). These findings suggest that BM-MSC may enhance thymic immune function through the upregulation of Tα1 and Tβ4 expression. BM-MSC also reduced systemic CRP, LBP, and LPS levels (Fig. 1F), which is consistent with prior reports [10]. Flow cytometry confirmed that BM-MSC normalized the proportions of cortical thymic epithelial cells (cTEC, Ly51^+^) and medullary thymic epithelial cells (mTEC, UEA1^+^) (Fig. 1G, gating strategy in Fig. S3). Functional assessment of TEC via MHC class II (a marker of epithelial maturation [25]) and Aire (an autoimmune regulator expressed by mTEC [25]) revealed increased MHC II expression in both mTEC and cTEC, alongside an elevated Aire^+^ cell frequency in mTEC (Fig. 1H). Thymic T-cell profiling analysis revealed greater proportions and numbers of double-positive (DP) cells in the BM-MSC group than in the Veh group, whereas the Veh group presented elevated CD8^+^ and double-negative (DN) T-cell ratios (Fig. 1I). Our study demonstrated that BM-MSC alleviate poststroke immunosuppression (PICS) by restoring TEC function to promote T-cell generation.

We assessed the temporal dynamics of peripheral immune cells and IL-10 levels from day 1–14 post tMCAO to identify the PICS. Our analysis revealed immunological trajectories. First, we observed a progressive decline in T-lymphocyte proportions, reaching a nadir on day 7 with incomplete recovery by day 14. Second, B-lymphocyte counts were minimized on day 3 before rebounding to near-baseline levels by day 14. Notably, neutrophil frequencies peaked acutely on day 3 prior to rapid decline, whereas monocyte populations exhibited progressive expansion that plateaued on day 7 and remained elevated through day 14. Most significantly, our cytokine profiling revealed that IL-10 levels peaked on day 7 and remained elevated through day 14 (Fig. S4A-E). We suggest that the confluence of five cardinal immunological manifestations on day 7 post stroke—the nadir of T-lymphocyte proportions, persistent B-lymphocyte suppression, resolving neutrophilia, monocytosis peak, and maximal IL-10 elevation—collectively designates this juncture as the immunological zenith of PICS.

To evaluate changes in peripheral lymphocytes, we performed flow cytometric analysis of the spleen and peripheral blood samples from 8-week-old mice on day 7 tMCAO. Similar to previous studies [26], we observed that, compared with Veh treatment, BM-MSC treatment partially restored the spleen weight (Fig. S5A). Histological evaluation via HE staining revealed preserved splenic architecture in the BM-MSC group (Fig. S5B). Flow cytometry analysis demonstrated that BM-MSC therapy significantly restored the proportions of total CD3^+^ T cells in the spleen and peripheral blood (Fig. S5C, F). Subgroup analysis further revealed increases in splenic and blood CD4^+^ T cells and CD8^+^ T cells (Fig. S5E, H). Notably, BM-MSC treatment did not significantly alter B-cell proportions (Fig. S5D, S5G). To assess therapeutic generalizability, we extended the investigations to aged tMCAO mice (18 months old). Stroke-induced reductions in T/B-cell ratios, phenocopying young mice, were observed in aged cohorts (Fig. S6A-H). BM-MSC effectively restored the peripheral and splenic T-cell proportions in aged mice. Intriguingly, BM-MSC significantly rescued splenic B-cell especially in aged animals, an effect that was not detected in young mice (Fig. S6F). Mechanistically, this age-dependent rescue may stem from exacerbated B-cell apoptosis in aged mice on day 7 post stroke, coupled with BM-MSC-mediated mitigation of caspase-1-dependent cell death in splenic marginal zone B cells, as previously reported [12].

Collectively, these findings suggest that BM-MSC counteract PICS by restoring thymic epithelial structure and function, thereby reconstructing T-cell developmental niches. This effect may be mediated via coordinated modulation of thymic hormone secretion and epithelial development.

### BM-MSC restore the atrophied thymus by promoting mTEC proliferation

**Fig. 2:**
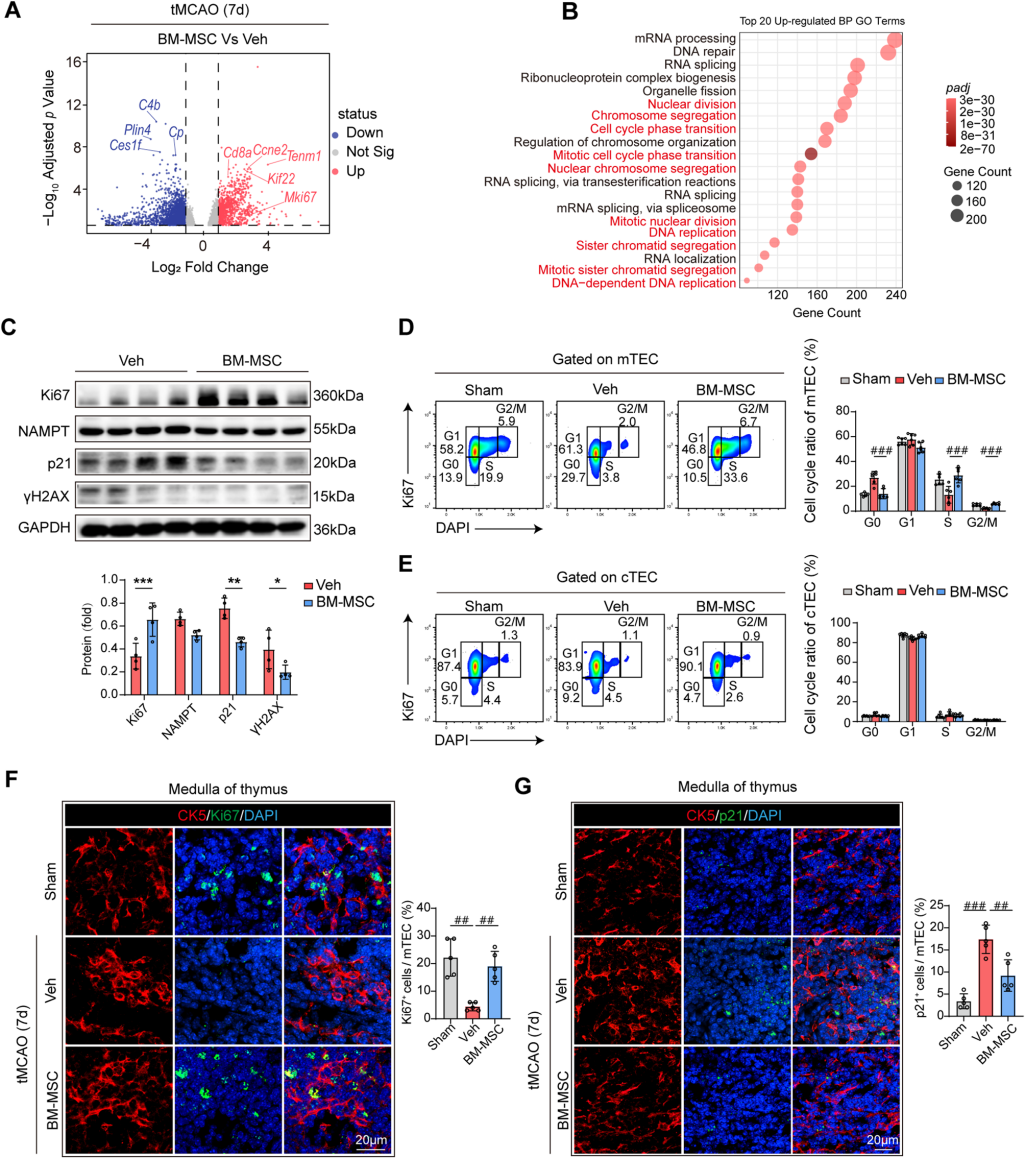
BM-MSC transplantation promotes the proliferation of medullary thymic epithelial cells (mTEC). Male wild-type C57/Bl6 mice underwent 60 minutes of tMCAO followed by reperfusion. BM-MSC (2 × 10⁶ cells/mouse) or vehicle (Veh) were administered intravenously as a single dose 2 hours postreperfusion. Thymic tissues were harvested at 7 days post-tMCAO. **A-B** Bulk RNA sequencing (RNA-seq) analysis of thymic tissues at 7 days post-tMCAO (*n* = 4 per group). **A** Volcano plot of differentially expressed genes (DEGs) between the BM-MSC and Veh groups. **B** Gene Ontology (GO) enrichment analysis of the top 20 enriched biological processes (BP) among the DEG. **C** Western blot analysis of proliferation-related (Ki67) and senescence-related (NAMPT, p21, and γH2AX) protein expression. The bar graphs show the relative protein levels (*n* = 4 per group). *p < 0.05, **p < 0.01, ***p < 0.001, by Student’ s *t*-test (mean ± SD). **D-E **Flow cytometry analysis of the cell cycle distribution of mTEC (EPCAM^+^CD45^−^UEA-1^+^Ly51^−^) **D** and cTEC (EPCAM^+^CD45^−^UEA-1^−^Ly51^+^) **E** costained with Ki67/DAPI. The bar graphs show the proportions of cells in the G0, G1, S, and G2/M phases (*n* = 6 per group). FDR-corrected *p*-values (*q-values)* were calculated, ###*q* < 0.001, one-way ANOVA (mean ± SD). **F** Immunofluorescence staining of the thymus: the medullary marker CK5 (red) and the proliferation marker Ki67 (green) are shown. Bar graphs showing Ki67^+^ cell proportions (*n* = 5 per group). FDR-corrected *p*-values (*q-values)* were calculated, ##*q* < 0.01, one-way ANOVA (mean ± SD). **G** Immunofluorescence staining of the thymus: the medullary marker CK5 (red) and the senescence marker p21 (green) are shown. The bar graphs show the proportions of p21^+^ cells (*n* = 5 per group). FDR-corrected *p*-values (*q-values)* were calculated, ##*q* < 0.01, ###*q* < 0.001, one-way ANOVA (mean ± SD)

To elucidate the mechanism underlying BM-MSC-mediated thymic regeneration, bulk RNA sequencing (RNA-seq) was performed on thymic tissues 7 days after tMCAO. Differential expression gene (DEG) analysis revealed significant upregulation of *Mki67* (a proliferation marker [27]) and *Cd8a* (a T-cell surface marker [28]) in BM-MSC-treated mice (Fig. 2A), indicating enhanced thymic proliferation and T-cell differentiation. GO enrichment analysis of the top 20 enriched biological processes in BM-MSC-treated mice highlighted proliferation-related pathways, including *nuclear division* and the *cell cycle phase transition* (Fig. 2B). Western blot analysis revealed increased Ki67 expression and reduced p21 and γH2AX levels in the BM-MSC group (Fig. 2C). On the basis of previous observations of BM-MSC-induced functional improvement in TEC and cortical–medullary ratio restoration, we hypothesized that BM-MSC promote TEC proliferation. Flow cytometry with Ki67/DAPI costaining revealed significantly greater proportions of mTEC in the S phase and G2/M phase in BM-MSC-treated mice, whereas Veh group mTEC predominantly remained in the G0 phase (Fig. 2D); however, cTEC exhibited no significant changes in the cell cycle (Fig. 2E). Immunofluorescence confirmed elevated Ki67 expression (Fig. 2F) and reduced p21 levels (Fig. 2G) in BM-MSC-treated mTEC. To confirm that mTEC restore thymic function following BM-MSC treatment, we evaluated T-cell receptor excision circles (TREC)—the gold standard for thymic output [29]—and conducted functional assessments of T cells in the thymus and peripheral blood (including IL-2/IFNγ production). TREC quantification confirmed enhanced thymic output in the BM-MSC groups (Fig. S7A). BM-MSC treatment significantly increased the number of IL-2⁺CD4⁺ T cells in the peripheral blood and thymus, increased the number of IFNγ⁺CD8⁺ T cells, and restored the number of peripheral IFNγ⁺CD4⁺ T cells to normal levels (Fig. S7B-D). Collectively, these data provide mechanistic evidence that BM-MSC-mediated reversal of thymic atrophy drives the proliferative rejuvenation of mTEC, enhances thymic output, and ultimately restores peripheral T-cell functional competence.

### BM-MSC promote TEC proliferation via migrasome-mediated translocation across the blood-thymus barrier

**Fig. 3:**
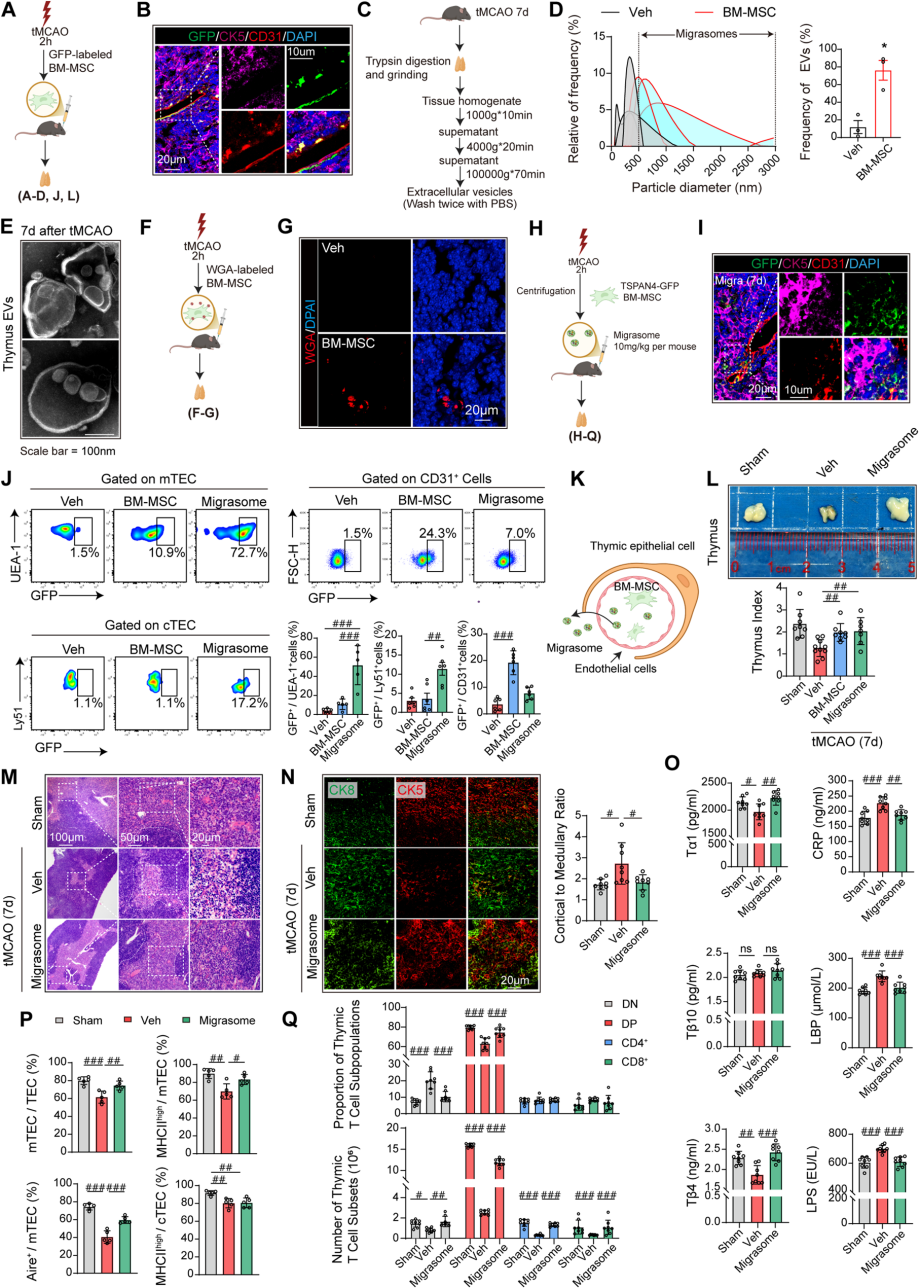
BM-MSC-derived migrasomes reverse poststroke thymic atrophy by traversing the BTB. Male wild-type C57/Bl6 mice underwent 60 minutes of tMCAO followed by reperfusion. **A-D**,** J**,** L** Schematic of the intravenous administration of GFP-labeled BM-MSC (2 × 10⁶ cells/mouse) or vehicle (Veh) 2 hours postreperfusion. **B** Representative immunofluorescence images of the thymus at 7 days post-tMCAO: GFP (green, BM-MSC), CK5 (purple), and CD31 (red, vascular marker). The experiments were repeated 3 times (*n* = 3 per group). **C** Schematic of thymic EVs isolation. **D** Size distribution analysis of EVs isolated from the Veh and BM-MSC groups (*n* = 3 per group). *p < 0.05 vs. Veh, by Student’ s *t*-test (mean ± SD). **E** Negative-staining transmission electron microscopy (TEM) images of thymic EVs from BM-MSC-treated mice. The experiments were repeated 3 times. **F-G** Schematic of WGA-labeled BM-MSC (2 × 10⁶ cells/mouse) or Veh administration **F** and representative thymic immunofluorescence images of WGA (red, migrasome marker) at 7 days post-tMCAO **G**. The experiments were repeated 3 times (*n* = 3 per group). **H-Q** Schematic of the intravenous administration of migrasomes (10 mg/kg/mouse, isolated from TSPAN4-GFP-overexpressing BM-MSC) or Veh **H**. **I** Immunofluorescence images of the thymus after migrasome monotherapy: GFP (green, migrasomes), CK5 (purple), and CD31 (red). The experiments were repeated 3 times. **J** Flow cytometry analysis of GFP signal proportions in mTEC, cTEC, and CD31^+^ cells from the Veh, BM-MSC, and migrasome groups (*n* = 6 per group). FDR-corrected *p*-values (*q-values)* were calculated, ##*q* < 0.01, ###*q* < 0.001, one-way ANOVA (mean ± SD). **K** Schematic illustrating that BM-MSC cannot cross the BTB, while their migrasomes penetrate the BTB. **L** Gross thymus images (*n* = 3 per group) and weight quantification (sham *n* = 8, Veh *n* = 10, BM-MSC *n* = 8, migrasome *n* = 7) on day 7. FDR-corrected *p*-values (*q-values)* were calculated, ##*q* < 0.01, one-way ANOVA (mean ± SD). **M** Representative H&E-stained thymic sections (*n* = 3 per group). **N** Immunofluorescence staining of the thymus: CK5 (red, medulla) and CK8 (green, cortex). The bar graphs show the cortical-to-medullary area ratios (*n* = 8 per group). FDR-corrected *p*-values (*q-values)* were calculated, #*q* < 0.05, one-way ANOVA (mean ± SD). **O** Plasma thymosin α1, β4, and β10 levels were measured via ELISA (*n* = 8 per group). Plasma CRP, LBP, and LPS levels were measured via ELISA (*n* = 8 per group). FDR-corrected *p*-values (*q-values)* were calculated, #*q* < 0.05, ##*q* < 0.01, ###*q* < 0.001, one-way ANOVA (mean ± SD). **P** Flow cytometry analysis of mTEC proportions, MHC II^+^ and Aire^+^ cells in mTEC, and MHC II^+^ cells in cTEC (bar graphs; *n* = 5 per group; flow cytometry diagram in Supplementary Fig. 7). FDR-corrected *p*-values (*q-values)* were calculated, #*q* < 0.05, ##*q* < 0.01, ###*q* < 0.001, one-way ANOVA (mean ± SD). **Q** Thymic T-cell subset profiling: proportions and counts of DN, DP, CD4^+^, and CD8^+^ T cells (*n* = 8 per group). FDR-corrected *p*-values (*q-values)* were calculated, #*q* < 0.05, ##*q* < 0.01, ###*q* < 0.001, one-way ANOVA (mean ± SD). A flow cytometry diagram is shown in Fig. S15

To investigate the mechanism by which BM-MSC promote mTEC proliferation, GFP-labeled BM-MSC were transplanted into tMCAO mice via lentiviral transduction (Fig. 3A). Immunofluorescence analysis of the thymus at 7 days post-tMCAO revealed predominant GFP signal colocalization with the CD31^+^ vasculature (CK5^+^, purple) in the thymic medulla, with minor vesicle-like GFP signals penetrating the medullary epithelium (Fig. 3B), suggesting that BM-MSC cannot directly traverse the blood-thymus barrier (BTB), but their secreted extracellular vesicles (EVs) may mediate this process. The therapeutic effects of BM-MSC involve multiple mechanisms, including EVs secretion and soluble factor release [30, 31]. Our previous research revealed that BM-MSC release migrasomes [11], organelles that form during cellular migration [32]. While monocyte-derived migrasomes have been shown to carry proangiogenic factors [33], whether BM-MSC-derived migrasomes target the thymus to regulate its regeneration remains unknown. We thus hypothesized that BM-MSC-derived migrasomes might deliver key regulatory factors across the BTB to restore thymic function and promote T-cell production.

To validate this hypothesis, EVs were isolated from the thymic tissues of tMCAO mice 7 days post-surgery (Fig. 3C). Size characterization revealed that thymic EVs were predominantly 500–3000 nm in size (Fig. 3D), which is consistent with reported migrasome dimensions [16]. Negative staining transmission electron microscopy (TEM) further confirmed the typical migrasome morphology—large vesicles with retraction fibers and internal vesicles (Fig. 3E). To track migrasome delivery, WGA-labeled BM-MSC (a migrasome-specific marker) were intravenously administered to tMCAO mice (Fig. 3F). Immunofluorescence analysis of thymic tissues 7 days post-tMCAO revealed WGA-positive migrasome-like structures (Fig. 3G). Collectively, these findings suggest that BM-MSC-derived migrasomes may represent a structural mechanism by which BM-MSC cross the BTB to restore thymic function and promote T-cell production.

To confirm the therapeutic role of migrasomes, migrasomes isolated from TSPAN4-GFP-overexpressing (a marker of migrasomes [34]) BM-MSC (Fig. S8A-D) were injected into tMCAO mice (Fig. 3H). TSPAN4 is a pivotal regulator of migrasome biogenesis [35], and TSPAN4 overexpression significantly increased migrasome production (Fig. S8G). Immunofluorescence analysis at 7 days post-tMCAO demonstrated specific accumulation of TSPAN4-GFP-labeled migrasomes in the thymic medulla without colocalization with the CD31^+^ vasculature (Fig. 3I). Flow cytometry revealed greater GFP signal intensity in mTEC and cTEC from migrasome-treated mice than in those from BM-MSC-transplanted mice, whereas BM-MSC-derived GFP signals were localized primarily to CD31^+^ cells (Fig. 3J). No significant GFP signals were detected in thymic T cells (CD3^+^), myeloid cells (CD11b^+^), or NK cells (NK1.1^+^) (Fig. S9), confirming the specificity of migrasome uptake by TEC (Fig. 3K).

To validate whether migrasomes can recapitulate the therapeutic effects of their parent cells, we further evaluated their therapeutic effects on thymic structure and function. Migrasome monotherapy (10 mg/kg) reversed thymic atrophy (Fig. 3L), restored the cortical-medullary architecture and thymic corpuscles (H&E staining; Fig. 3M), and normalized the cortical-medullary ratios (Fig. 3N). Migrasome treatment elevated plasma thymosin α1 and β4 levels but had no significant effect on the Tβ10 concentration; reduced CRP, LBP, and LPS levels (Fig. 3O); increased mTEC proportions; and increased MHC II^+^ and Aire^+^ cells in mTEC, whereas cTEC MHC II expression remained unchanged (Fig. 3P). Thymic T-cell profiling revealed increased DP cell proportions and numbers, reduced DN ratios, and elevated CD4^+^ and CD8^+^ T-cell numbers in migrasome-treated mice (Fig. 3Q). These data indicate that migrasome therapy can effectively recapitulate the thymic regenerative effects of parent BM-MSC.

We further explored the systemic immunomodulatory effects of migrasomes. Migrasome therapy also restored the spleen index and architecture (Fig. S5A, B). Flow cytometry analysis revealed increased CD3^+^ T-cell proportions in the spleen and peripheral blood (Fig. S5C, F), with elevations in splenic CD8^+^ and CD4^+^ T cells and peripheral blood CD8^+^ and CD4^+^ T cells (Fig. S5E, H).

Notably, we confirmed that the therapeutic benefits of migrasomes—including thymic regeneration and systemic T-cell reconstitution—were consistently observed in female mice, demonstrating that the efficacy is sex-independent (Fig. S10).

Additionally, we investigated the neuroprotective effects of BM-MSC-derived migrasomes. Migrasome treatment improved survival and neurological scores (Fig. S1D, E), reduced cerebral infarct volume (Fig. S1F), restored white matter integrity (Fig. S1G), and reduced immune cell infiltration (Fig. S2). To further evaluate the neurorestorative effects of migrasomes, functional assessments were conducted via established behavioral paradigms. The migrasome-treated group presented enhanced motor coordination, as evidenced by the rotarod test (Fig. S11B), and improved limb function, as shown by the foot fault assay (Fig. S11C). This group subsequently demonstrated significant improvements in cognitive functions, including novel object recognition capacity, as assessed by standardized testing (Fig. S11D), and spatial learning/memory, as validated by the Morris water maze (Fig. S11E-F).

Collectively, these findings demonstrate that BM-MSC-derived migrasomes can cross the blood-thymus barrier (BTB) to directly target mTEC, thereby reconstructing T-cell developmental niches and recapitulating the thymic regenerative effects of parent BM-MSC. This mechanism alleviates PICS. Importantly, BM-MSC-derived migrasomes also exhibit direct neuroprotective effects, suggesting their potential as a novel therapeutic strategy for stroke recovery.

### BM-MSC-derived migrasomes recapitulate the therapeutic mechanisms of parental cells

**Fig. 4:**
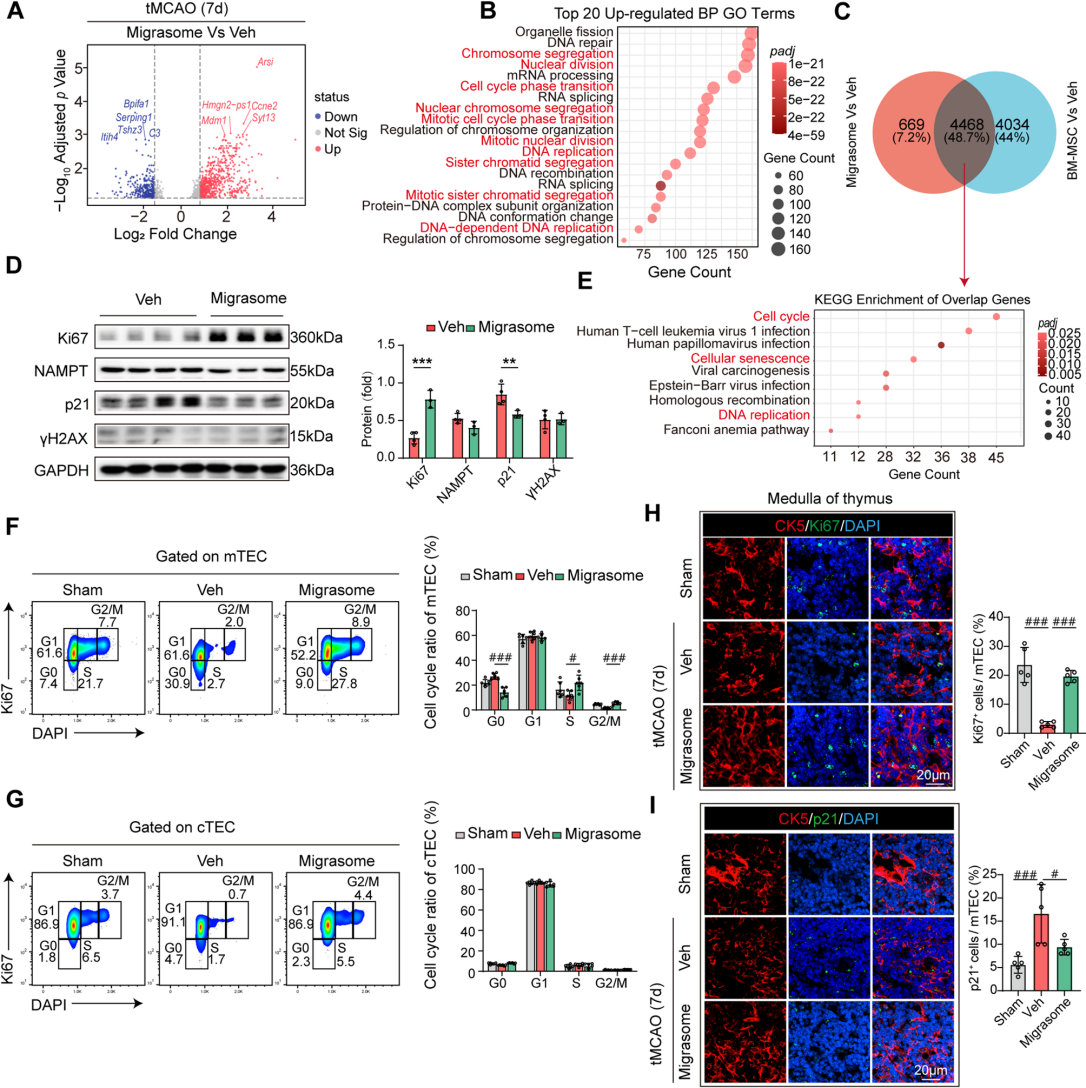
BM-MSC-derived migrasomes recapitulate parental cell-induced mTEC proliferation. Male wild-type C57/Bl6 mice underwent 60 minutes of tMCAO followed by reperfusion. Migrasomes (10 mg/kg/mouse) isolated from TSPAN4-GFP-overexpressing BM-MSC or vehicle (Veh) were administered intravenously as a single dose 2 hours postreperfusion. Thymic tissues were harvested at 7 days post-tMCAO. **A-C**,** E** Bulk RNA sequencing (RNA-seq) analysis of thymic tissues (*n* = 4 per group). **A** Volcano plot of DEGs between the Migrasome and Veh groups. **B** GO enrichment analysis of the top 20 upregulated BPs from the DEG. **C** Venn diagram showing overlapping DEGs between the Migrasome and Veh groups and between the BM-MSC and Veh groups. **D** Western blot analysis of proliferation-related (Ki67) and senescence-related (NAMPT, p21, and γH2AX) protein expression. The bar graphs show the relative protein levels (Veh *n* = 4, Migrasome *n* = 3). **p < 0.01, ***p < 0.001, by Student’ s *t*-test (mean ± SD). **E** KEGG pathway enrichment analysis of the overlapping DEGs. **F-G** Flow cytometry analysis of the cell cycle distribution of mTEC **F** and cTEC **G** costained with Ki67/DAPI. The bar graphs show the proportions of cells in the G0, G1, S, and G2/M phases (*n* = 6 per group). FDR-corrected *p*-values (*q-values)* were calculated, #*q* < 0.05, ###*q* < 0.001, one-way ANOVA (mean ± SD). **H** Immunofluorescence staining of the thymus: the medullary marker CK5 (red) and the proliferation marker Ki67 (green) are shown. Bar graphs showing Ki67^+^ cell proportions (*n* = 5 per group). FDR-corrected *p*-values (*q-values)* were calculated, ###*q* < 0.001, one-way ANOVA (mean ± SD). **I** Immunofluorescence staining of the thymus: the medullary marker CK5 (red) and the senescence marker p21 (green) are shown. The bar graphs show the proportions of p21^+^ cells (*n* = 5 per group). FDR-corrected *p*-values (*q-values)* were calculated, #*q* < 0.05, ###*q* < 0.001, one-way ANOVA (mean ± SD)

To investigate the mechanism underlying migrasome-mediated restoration of poststroke thymic atrophy, bulk RNA sequencing (RNA-seq) was performed on thymic tissues from Migrasome-treated mice 7 days post-tMCAO. DEG analysis revealed significant upregulation of the expression of *Ccne2* (a cell cycle regulator [36]) in the migrasome group (Fig. 4A), indicating increased proliferative activity in the atrophied thymus. GO enrichment analysis of the top 20 enriched biological processes highlighted proliferation-associated pathways, including *chromosome segregation* and *mitotic nuclear division* (Fig. 4B). Comparative analysis of DEGs between the Migrasome/Veh and BM-MSC/Veh groups revealed 48.7% overlap (Fig. 4C), with shared DEGs significantly enriched in pathways such as *the cell cycle*, *cellular senescence*, and *DNA replication* (Fig. 4E). Western blot analysis revealed increased Ki67 expression and reduced p21 levels in the migrasome group, whereas NAMPT and γH2AX levels remained unchanged (Fig. 4D). Flow cytometry with Ki67/DAPI costaining confirmed greater proportions of mTEC in the S phase and G2/M phase in migrasome-treated mice than in Veh-treated mice (Fig. 4F), whereas cTEC presented no significant alterations in the cell cycle (Fig. 4G). Immunofluorescence further revealed elevated Ki67 expression and reduced p21 levels in migrasome-treated mTEC (Fig. 4H, I). Quantitative analysis of TRECs revealed that migrasome treatment also enhanced thymic output function, recapitulating the improvement in thymic and peripheral T cell function mediated by BM-MSC (Fig. S7). These findings collectively establish that BM-MSC-derived migrasomes recapitulate the therapeutic mechanisms of their parental cells, suggesting that migrasomes primarily mediate BM-MSC-induced thymic regeneration.

### ScRNA-seq reveals BM-MSC and migrasome-mediated expansion and enhanced developmental progression of the mTECⅠ subpopulation

**Fig. 5:**
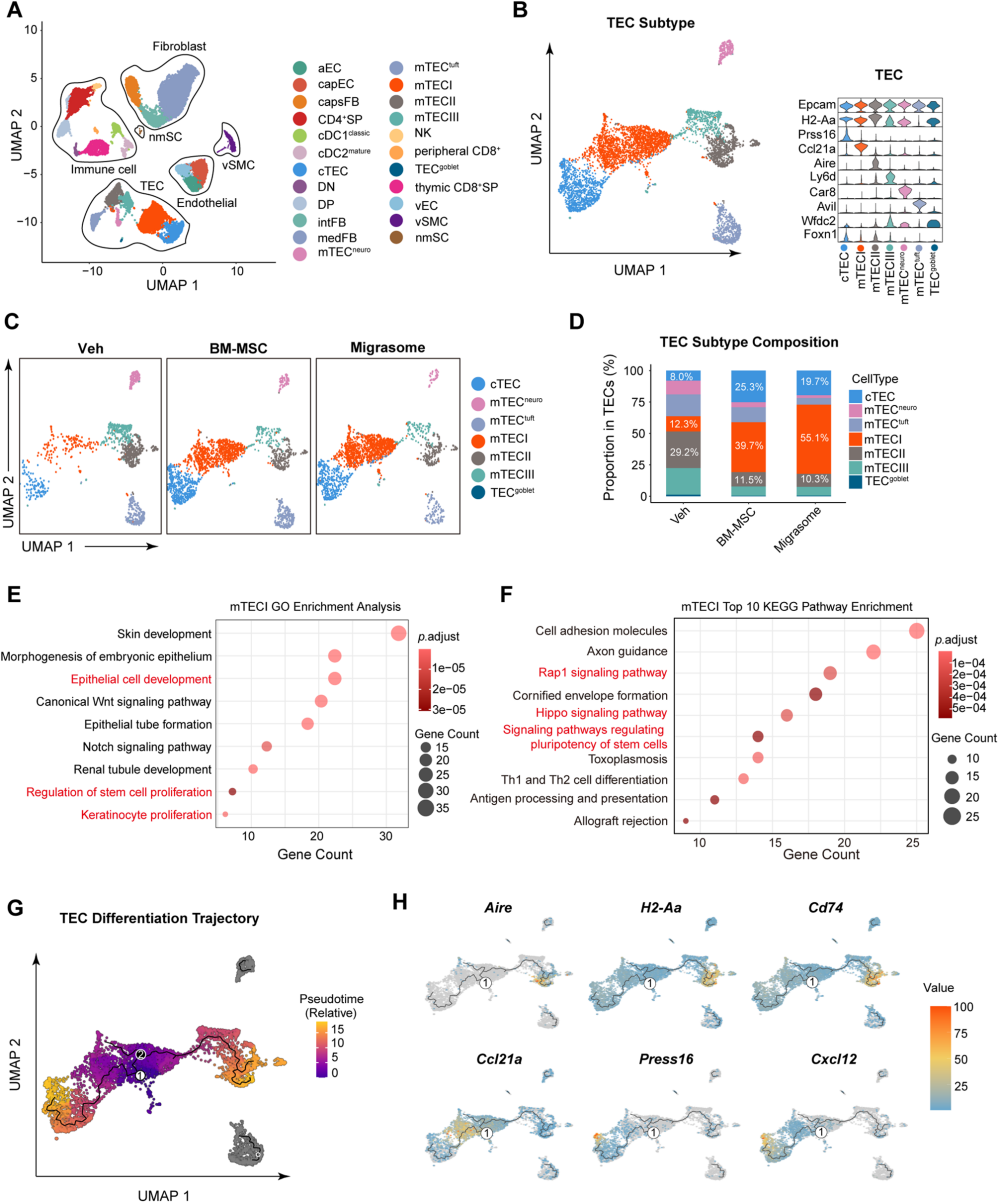
ScRNA-seq reveals BM-MSC and migrasome-mediated expansion and enhanced developmental progression of mTECⅠ subpopulation. Male wild-type C57/Bl6 mice underwent 60 min of tMCAO followed by reperfusion. Migrasomes (10 mg/kg/mouse) isolated from TSPAN4-GFP-overexpressing BM-MSC or vehicle (Veh) were administered intravenously as a single dose 2 h postreperfusion. Thymic tissues were harvested at 7 days post-tMCAO. **A** Uniform manifold approximation and projection (UMAP) projection of 21,562 thymic cells (17,396 CD45⁻ + 4,166 CD45⁺) from post-stroke mice. Major compartments annotated: TEC, immune cell, fibroblast, endothelial, vascular smooth muscle cell, and non-myelinating Schwann cell. Solid lines demarcate compartment boundaries. **B** TEC subclustering: (Left) Re-analyzed UMAP of TEC subtype; (Right) Violin plots showing marker gene expression. **C** UMAP of TEC across Veh, BM-MSC, and Migrasome groups. **D** Stacked bar plot depicting proportional changes of TEC subtype. **E** GO enrichment of proliferation/development pathways in mTECⅠ. **F** Top 10 KEGG pathways enriched in mTECⅠ. **G** Pseudotemporal trajectory analysis via Monocle3: TEC developmental progression (pseudotime value increases from purple to yellow). mTECⅠ localizes to trajectory origin (lowest pseudotime). **H** Dynamic expression of key differentiation regulators along trajectories: *Aire*/*H2-Aa*/*Cd74* (mTECⅡ); *Ccl21a*/*Prss16*/*Cxcl12* (cTEC)

To comprehensively resolve post-treatment changes in thymic cell populations, we performed single-cell RNA sequencing (scRNA-seq) on thymus tissues from the Veh, BM-MSC, and Migrasome- treated groups of mice. To capture the relatively rare TEC more effectively, we employed CD45- based enrichment sorting of murine thymocytes. Specifically, the cells were sorted at a ratio of CD45^−^:CD45^+^ = 5:1, resulting in 17,396 CD45^−^ cells and 4,166 CD45^+^ cells, which were pooled for scRNA-seq. On the basis of marker genes from published thymic scRNA-seq datasets [37], we annotated the major cellular populations as follows: TEC(*Epcam*^+^, *H2-Aa*^+^), immune cell (*Ptprc*^+^), endothelial cell (EC, *Pecam1*^+^, *Cdh5*^+^), fibroblast (FB, *Pdgfra*^+^), vascular smooth muscle cells (vSMC, *Acta2*^+^, *Myl9*^+^), and rare non-myelinating Schwann cells (nmSC, *Gfap*^+^, *S100b*^+^) (Fig. 5A).

Leveraging ThymoSight (www.thymosight.org), we refined the subpopulations within each lineage (Fig. S12A). To investigate BM-MSC and Migrasome-mediated effects on post-stroke TEC, we subsampled and reanalyzed TEC clusters (Fig. 5B), categorizing them into seven subsets: cortical TEC (cTEC, high *Prss16*), medullary TEC I (mTECⅠ, high *Ccl21a*), medullary TEC II (mTECⅡ, high *Aire*), mTECⅢ (high *Ly6d*), neuroendocrine-like TEC (TEC^neuro^, high *Car8*), tuft-like TEC (TEC^tuft^, high *Avil*), and goblet cell-like TEC (TEC^goblet^, high *Wfdc2*) (Fig. 5B).

Comparative analysis of thymic cellular composition across the Veh, BM-MSC-, and Migrasome-treated groups via UMAP visualization revealed the most pronounced proportional changes in mTECⅠ, cTEC, and DP thymocytes (Fig. S12B). Among them, both the BM-MSC and Migrasome groups exhibited significantly higher mTECⅠ and cTEC counts than the Veh group (Fig. 5C). GO enrichment analysis of the mTECⅠ population revealed significant enrichment of pathways related to “Epithelial cell development”, “Regulation of stem cell proliferation”, and “Keratinocyte proliferation” (Fig. 5E). Complementary KEGG analysis further revealed upregulation of the “Rap1 signaling pathway”, “Hippo signaling pathway”, and “Signaling pathways regulating pluripotency of stem cells” (Fig. 5F). These findings collectively suggest that therapeutic interventions primarily restore overall mTEC homeostasis through mTECⅠ expansion. Furthermore, analysis of immune cell subpopulations revealed a significant increase in the proportion of DP cells within the thymus in the BM-MSC and Migrasome groups (Fig.S12C). The observed changes in TEC and immune cell subpopulations align well with our flow cytometry results, which indicated that both BM-MSC and Migrasome treatments promote mTEC proliferation and maintain central immune homeostasis.

To delineate the mTECⅠ differentiation potential, pseudotemporal trajectory analysis via *Monocle3* assigned mTECⅠ with lowest pseudotime values, which is indicative of an early developmental state (Fig. 5G). The trajectory indicates the potential of mTECⅠ to differentiate into both cTEC and mTECⅡ. We subsequently analyzed the dynamic expression of DEGs along the inferred trajectories. Key regulatory genes representative of the mTECⅠ-to-mTECⅡ differentiation (e.g., *Aire*, *H2-Aa*, *Cd74*) and the mTECⅠ-to-cTEC differentiation (e.g., *Ccl21a*, *Prss16*, *Cxcl12*) were identified (Fig. 5H). The expression patterns of these genes clearly delineate the developmental trajectories from mTECⅠ to cTEC and mTECⅡ. Therefore, our scRNA-seq data indicate that BM-MSC and Migrasome treatments may contribute to the maintenance of central immune homeostasis by promoting the proliferation and subsequent differentiation of mTECⅠ.

### Inhibition of migrasome production in BM-MSC abrogates their therapeutic efficacy in poststroke thymic atrophy

**Fig. 6:**
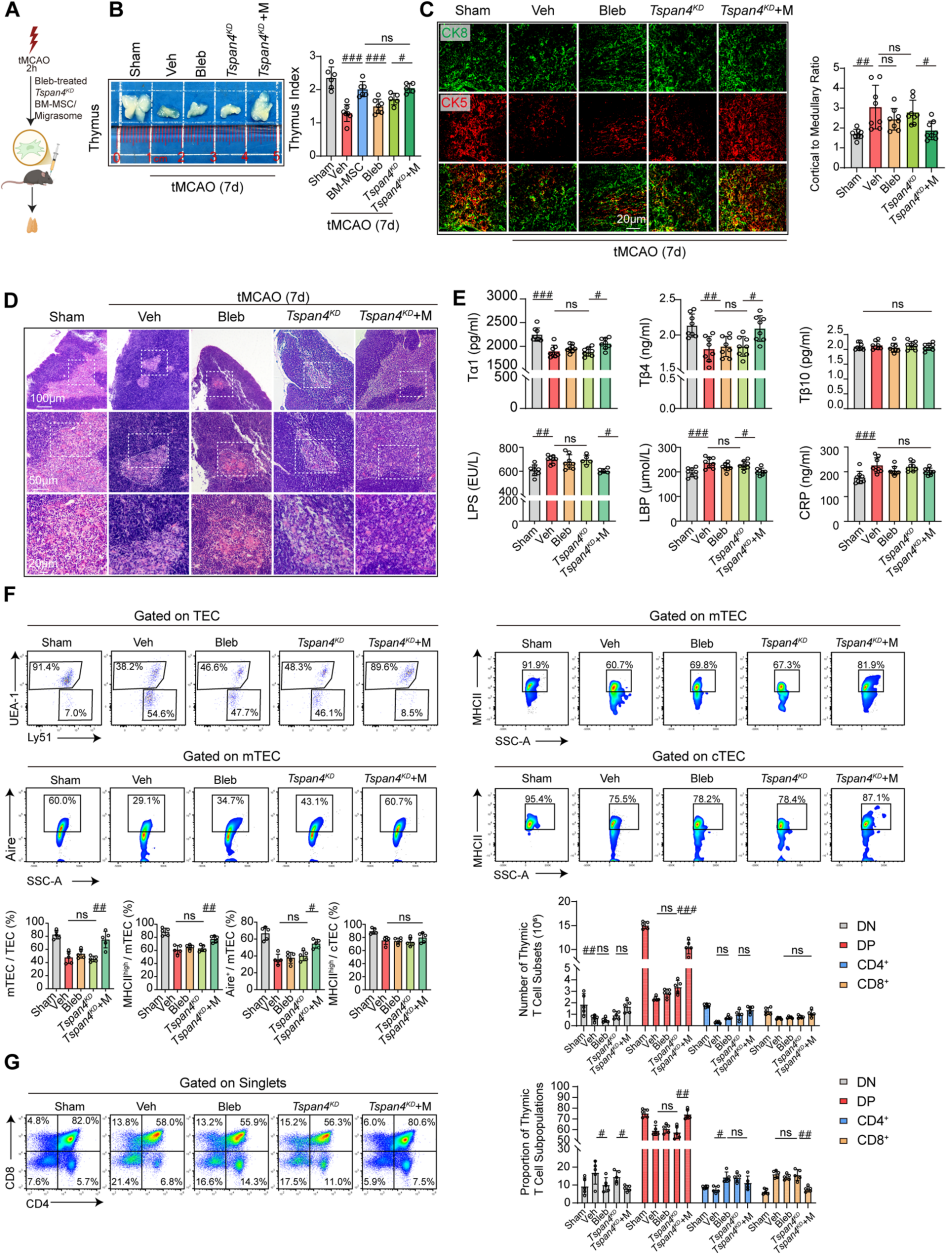
Inhibition of migrasome production in BM-MSC abrogates their therapeutic effects on poststroke thymic atrophy. Male wild-type C57/Bl6 mice underwent 60 min of tMCAO followed by reperfusion. **A** Schematic illustrating the intravenous administration of Vehicle (Veh), blebbistatin-pretreated BM-MSC (Bleb), *Tspan4*^KD^ BM-MSC (*Tspan4*^KD^), or *Tspan4*^KD^ BM-MSC with WT migrasome add-back (*Tspan4*^KD^ + M), all performed at 2 h post-reperfusion.The mice were sacrificed at 7 days post-tMCAO. **B** Gross thymus images (*n* = 3 per group) and weight quantification (sham *n* = 6, Veh *n* = 7, BM-MSC *n* = 6, Bleb *n* = 7, *Tspan4*^KD^ *n* = 5, *Tspan4*^KD^ + M *n* = 5) on day 7. FDR-corrected *p*-values (*q-values*) were calculated, #*q* < 0.05, ###*q* < 0.001, one-way ANOVA (mean ± SD). **C** Immunofluorescence staining of the thymus: CK5 (red, medulla) and CK8 (green, cortex). The bar graphs show the cortical-to-medullary area ratios (*n* = 8 per group). FDR-corrected *p*-values (*q-values*) were calculated, #*q* < 0.05, ##*q* < 0.01, one-way ANOVA (mean ± SD). **D** Representative H&E-stained thymic sections (*n* = 3 per group). **E** Plasma thymosin α1, β4, β10 and LPS, LBP, CRP levels were measured via ELISA (*n* = 8 per group). FDR-corrected *p*-values (*q*-values) were calculated, #*q* < 0.05, ##*q* < 0.01, ###*q* < 0.001, one-way ANOVA (mean ± SD). **F** Flow cytometric analysis of TEC. Graphs show the proportions of mTEC and cTEC, and the frequencies of MHC II^+^ or Aire^+^ cells within mTEC and MHC II^+^ cells within cTEC (*n* = 5 mice per group). FDR-corrected *p*-values (*q-values*) were calculated, #*q* < 0.05, ##*q* < 0.01, one-way ANOVA (mean ± SD). **G** Thymic T-cell subset profiling: proportions and counts of DN, DP, CD4^+^, and CD8^+^ T cells (*n* = 5 per group). FDR-corrected *p*-values (*q-values*) were calculated, #*q* < 0.05, ##*q* < 0.01, one-way ANOVA (mean ± SD)

To confirm the dependency of BM-MSC-mediated thymic repair on migrasomes, BM-MSC were treated with blebbistatin (Bleb) to inhibit migrasome generation and intravenously (2 × 10^6^ cells/mouse) administered to tMCAO mice. Bleb can be targeted to inhibit myosin so that cells cannot migrate [17], thereby reducing the production of migrasomes (Fig. S13B). We also established a *Tspan4*-knockdown (*Tspan4*^KD^) BM-MSC cell line, which similarly exhibited reduced migrasome production (Fig. S8E-G). The therapeutic efficacy was then compared across five experimental groups: Sham, Vehicle (Veh), Bleb-pretreated BM-MSC (Bleb), *Tspan4*^KD^ BM-MSC (*Tspan4*^*KD*^), and *Tspan4*^KD^ BM-MSC co-administered with exogenous WT BM-MSC-derived migrasomes (*Tspan4*^KD^ + M) (Fig. 6A). Both Bleb-pretreated and *Tspan4*^KD^ BM-MSC failed to reverse thymic atrophy (Fig. 6B) or restore the disordered corticomedullary architecture, as shown by H&E staining and immunofluorescence (Fig. 6C, D). Crucially, the co-administration of exogenous WT migrasomes rescued the thymic index and structural defects induced by *Tspan4*^KD^ (Fig. 6B-D). ELISA indicated that both Bleb and *Tspan4*^KD^ treatments significantly decreased plasma thymosin-α1 and -β4 versus Sham, without ameliorating inflammatory markers (LPS, LBP, CRP) versus Veh (Fig. 6E). In contrast, WT migrasome add-back specifically restored thymosin levels and suppressed LPS and LBP (Fig. 6E).

Flow cytometry revealed that neither Bleb-pretreated nor *Tspan4*^KD^ BM-MSC increased mTEC proportions, nor did they elevate the frequencies of MHC II⁺ or Aire⁺ cells in mTEC or MHC II⁺ cells in cTEC (Fig. 6F). Notably, the co-administration of exogenous WT migrasomes significantly rescued these deficiencies resulting from impaired migrasome production, thereby demonstrating the functional specificity of migrasomes in T-cell reconstitution (Fig. 6F, G).

Collectively, these data indicate that BM-MSC with impaired migrasome production, whether through pharmacological inhibition (Bleb) or genetic knockdown (*Tspan4*^KD^), cannot restore thymic structure or function in tMCAO mice. Most importantly, the efficacy of *Tspan4*^KD^ BM-MSC was restored by exogenous migrasome add-back, providing evidence that migrasomes are the essential component mediating the thymic regenerative effects of BM-MSC.

Given that *Tspan4*^KD^ and Bleb-pretreated BM-MSC produced highly concordant phenotypes in thymic repair, subsequent evaluations of systemic immunity and neurological function were conducted using the Bleb model. Consistent with these previous findings, we further evaluated the systemic immunomodulatory effects. Bleb-treated BM-MSC failed to restore the spleen index or architecture (Fig. S5A, B). Flow cytometry analysis revealed no improvement in splenic or peripheral blood CD3^+^ T-cell proportions (Fig. S5C, F). Quantitative analysis of TRECs and functional assessment of T cells also revealed that the Bleb-treated group failed to recapitulate the BM-MSC-mediated increase in thymic output and improvement in T cell function (Fig. S7). These results confirm that migrasome deficiency abrogates BM-MSC-mediated thymic repair and peripheral immune modulation, thereby establishing migrasomes as critical effector components of BM-MSC therapy in stroke recovery.

Notably, Bleb-treated BM-MSC reduced the cerebral infarct volume, preserved white matter integrity (Fig. S1F, G), and improved motor function (rotarod; foot fault) (Fig. S11B, C), but there was no significant difference in the survival rate between Veh (Fig. S1D). In the Morris water maze test, Bleb-treated mice presented a shorter latency without enhanced target annulus crossovers or time in the target quadrant (Fig. S9E, F). Intracranial immune profiling revealed decreased numbers of macrophages and DN T cells but no significant changes in total T cells, B cells, neutrophils (Ly6G^+^), microglia (CD11b^+^CD45^−^), or CD8^+^ or CD4^+^ T cells (Fig. S2). These findings collectively demonstrate that Bleb-treated BM-MSC retain partial neuroprotective effects but fail to restore thymic architecture or promote T-cell differentiation due to migrasome deficiency, resulting in attenuated overall therapeutic efficacy.

### BM-MSC-derived migrasomes promote mTEC proliferation via Pin1 protein transfer

**Fig. 7:**
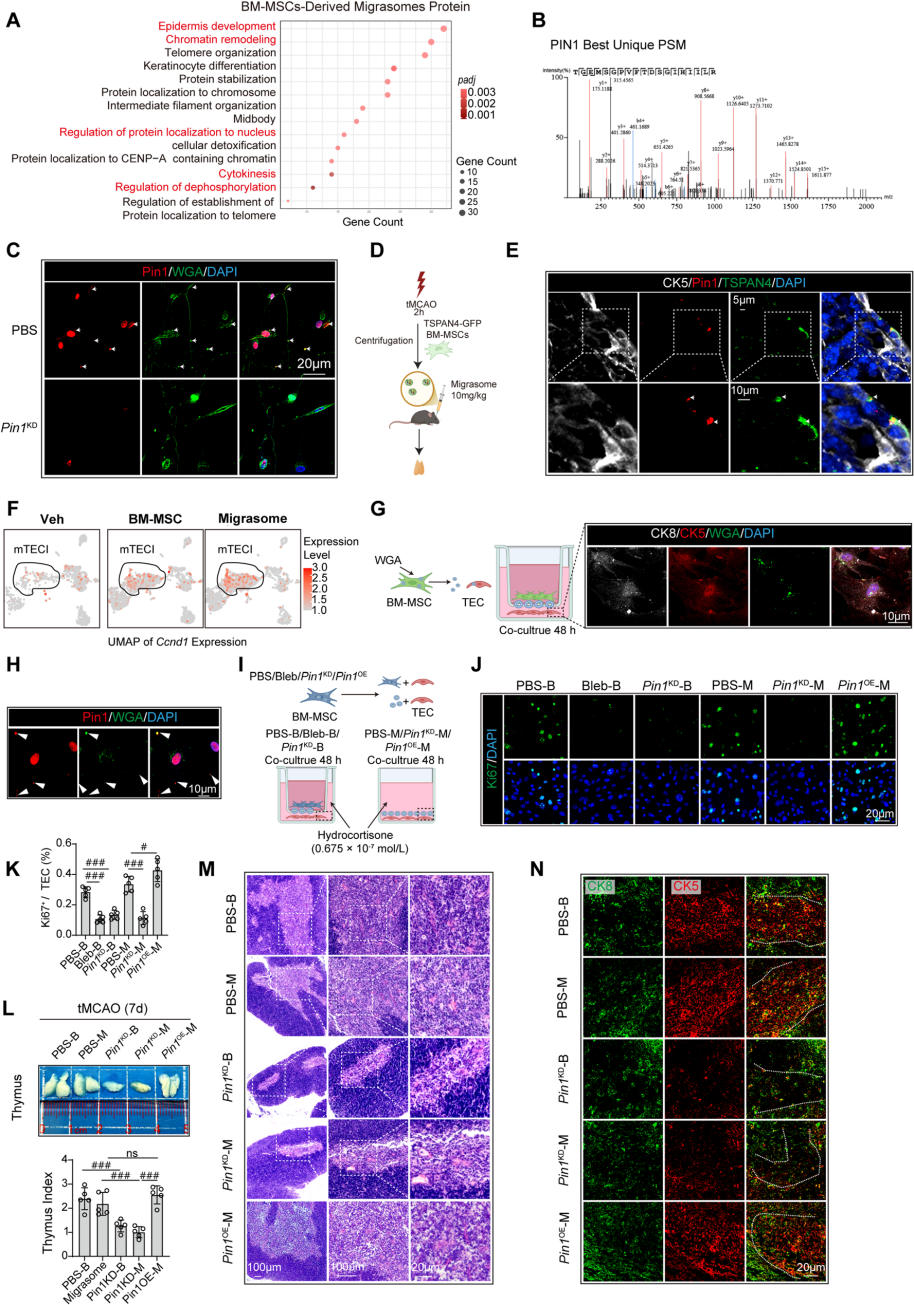
BM-MSC-derived migrasomes promote mTEC proliferation via Pin1 protein transfer. **A** Isolation of BM-MSC-derived migrasomes and liquid chromatography‒mass spectrometry (LC‒MS) proteomic analysis, with GO enrichment of identified proteins. **B** Protein coverage and peptide spectrum match (PSM) of Pin1 in migrasomes. **C **Co-staining of Pin1 (red) and WGA (green, migrasome marker) in PBS or P*in1*^KD^ treated BM-MSC cultured on fibronectin-coated coverslips for 6 h. The experiments were repeated three times; representative images are shown. **D** Schematic of the intravenous administration of migrasomes (10 mg/kg/mouse) isolated from TSPAN4-GFP-overexpressing BM-MSC. **E** Immunofluorescence of the thymus at 7 days post-tMCAO: CK5 (white, medulla), Pin1 (red), and TSPAN4 (green). The experiments were repeated three times; representative images are shown. **F** sc-RNAseq-derived TEC UMAP heatmaps showing *Ccnd1* expression across groups. **G** Transwell coculture design: Wild-type neonatal TECs (lower chamber) were cocultured with WGA-labeled BM-MSC (upper chamber, 5:1 ratio) for 48 h (1:1 medium mixture). TECs were stained with CK5 (red, TEC marker) and CK8 (white, TEC marker). The experiments were repeated three times; representative images are shown. **H** Pin1 (red) staining of TECs from transwell cocultures. The white arrows indicate WGA (green) and Pin1 colocalization. The experiments were repeated three times; representative images are shown. **I** Experimental schematics: (Bottom left) Transwell co-culture: PBS/Bleb/*Pin1*^KD^ pretreated BM-MSC with TEC (1:5 ratio) in 0.675 µM hydrocortisone-containing medium for 48 h. (Bottom right) Direct migrasome treatment: Migrasomes (10 µg/ml) isolated from PBS/*Pin1*^KD^/*Pin1*^OE^ BM-MSC co-cultured with TEC for 48 h (0.675 µM hydrocortisone). **J**,** K** Ki67 immunofluorescence in TEC and its quantification. Representative images are shown in **J**. The proportion of Ki67⁺ TEC was quantified in **K** (*n* = 5). Data represent mean ± SD; *n* = 5 per group. FDR-corrected *p*-values (*q-values*) were calculated, #*q* < 0.05, ###*q* < 0.001, by one-way ANOVA (mean ± SD). Representative images are shown. **L** Gross thymic morphology and thymic index at day 7. Data represent mean ± SD; *n* = 5 per group. FDR-corrected *p*-values (*q-values*) were calculated, ###*q* < 0.001, by one-way ANOVA (mean ± SD). **M** H&E staining showing corticomedullary architecture. **N** Immunofluorescence for CK5 (red, medullary marker) and CK8 (green, cortical marker). Dashed lines demarcate cortex and medulla

Previous studies have demonstrated that migrasomes serve as carriers of organelles and functional protein components [38, 39], playing pivotal roles in cell‒cell communication [40]. Building upon this foundation, we hypothesized that BM-MSC-derived migrasomes might mediate proproliferative effects through specific protein cargo. To test this hypothesis, proteomic analysis of BM-MSC-derived migrasomes was conducted via liquid chromatography‒tandem mass spectrometry (LC‒MS/MS). GO enrichment analysis revealed significant enrichment of Migrasome proteins in pathways related to *epidermis development*, *regulation of protein localization to the nucleus*, and *cytokinesis* (Fig. 7A), suggesting their role in promoting proliferation. Notably, the cell cycle regulator Pin1 (peptidyl-prolyl cis-trans isomerase NIMA-interacting 1), which drives cell cycle progression via interactions with cyclin-dependent kinases [41], was identified (Fig. 7B). Pin1 was previously reported to localize primarily to the nucleus and has not been detected in EVs or migrasomes [42].

To determine whether Pin1 mediates the therapeutic effects of BM-MSC-derived migrasomes, we generated *Pin1*-knockdown (*Pin1*^KD^) BM-MSC via shRNA interference (Fig. S8H) and tracked Pin1 protein expression both in vitro and in vivo. In vitro experiments confirmed the presence of Pin1 in WGA-labeled migrasomes derived from PBS treated BM-MSC, whereas the migrasomes produced by *Pin1*^KD^ BM-MSC completely lacked Pin1 signals (Fig. 7C). In vivo, GFP-tagged Pin1 could be detected within mTEC in tMCAO mice injected with migrasomes isolated from TSPAN4-GFP-overexpressing BM-MSC (Fig. 7D, E). Interestingly, scRNA-seq demonstrated significantly elevated expression of *Ccnd1*, a key *Pin1* downstream effector [43], in mTECⅠ clusters, showing Migrasome >BM-MSC >Veh hierarchy (Fig. 7F). These findings suggest that Pin1 may represent a critical therapeutic factor within BM-MSC-derived migrasomes.

To further validate this hypothesis, Transwell coculture experiments demonstrated that WGA-positive migrasomes containing Pin1 were internalized by TEC after 48 h of coculture with BM-MSC (WGA-labeled, green) (Fig. 7G, H). In a hydrocortisone-induced TEC senescence model (0.675 µM), both PBS-treated BM-MSC (via Transwell co-culture) and isolated migrasomes (direct treatment) rescued TEC proliferative capacity (2–3 fold increase in Ki67⁺ cells), effects fully abrogated by Bleb or *Pin1*^KD^ (Fig. 7I-K). Importantly, in vivo, *Pin1*^KD^ BM-MSC or their migrasomes failed to restore thymic architecture, resulting in a lower thymic index versus PBS treatments and disrupted corticomedullary demarcation, as shown by histomorphometric analysis via H&E or CK5/CK8 staining (Fig. 7L-N).

To directly test the sufficiency of Pin1, we generated Pin1-overexpressing BM-MSC (Fig. S8I) and isolated their migrasomes (*Pin1*^OE^-M). In the hydrocortisone-induced senescence model, *Pin1*^OE^-M significantly enhanced the proportion of Ki-67⁺ TEC compared to control migrasomes (Fig. 7I-K). In tMCAO mice, *Pin1*^OE^-M administration induced a restorative trend in the thymic index and yielded significantly better outcomes than *Pin1*-knockdown treatments, with histological analyses confirming a clear rescue of thymic architecture (Fig. 7L-N).

Collectively, these data demonstrate that BM-MSC-derived migrasomes deliver Pin1 to TEC, thereby promoting their proliferation and development. Through combined loss-of-function (*Pin1*^KD^) and gain-of-function (*Pin1*^OE^) approaches, we establish that Pin1 within migrasomes is both necessary and sufficient for mediating these therapeutic effects. This mechanism likely explains how BM-MSC restore thymic structure and function in tMCAO mice.

## Discussion

The current study reveals a novel therapeutic mechanism by which BM-MSC-derived migrasomes alleviate poststroke immunosuppression through targeted delivery of the Pin1 protein to TEC, thereby reversing stroke-induced thymic atrophy and restoring peripheral immune homeostasis. This work not only advances our understanding of migrasome biology but also provides a cell-free strategy to address the clinical challenge of balancing neuroprotection and immune restoration after ischemic stroke.

We demonstrate for the first time that BM-MSC utilize migrasomes to cross the BTB and act on mTEC, promoting their proliferation and reconstructing T-cell developmental niches. Mechanistically, integrated RNA-seq and proteomic analyses identified Pin1—a cell cycle regulator—as a key cargo protein within migrasomes that drives mTEC proliferation. Crucially, the inhibition of migrasome production (e.g., via Bleb and *Tspan4*^KD^) completely abrogated the thymic regenerative effects of BM-MSC, confirming that migrasomes are essential therapeutic vehicles. Furthermore, migrasome monotherapy not only recapitulates BM-MSC-mediated thymic regeneration but also significantly improves neurological outcomes. However, when BM-MSC are inhibited from producing migrasomes through Bleb, the inherent ability of parent cells to improve neuroinflammation and cognitive function is partially suppressed. These findings indicate that migrasomes have multitarget synergistic effects. Notably, the detection of nuclear localization proteins within migrasomes implies potential previously unrecognized nucleus–migrasome crosstalk.

This study addresses a critical gap in stroke management. Current therapies targeting the SNS or HPA axis risk disrupting endogenous neuroprotective pathways [6], whereas migrasome therapy achieves dual benefits of neuroprotection (e.g., reduced infarct size, improved motor/cognitive function) and immune restoration (thymic regeneration, T-cell reconstitution). Migrasomes are vesicular structures (diameter: 0.5–3 μm) formed and released at the ends of retraction fibers left by migrating cells [39]. They contain abundant intraluminal vesicles capable of transporting proteins, mRNAs, and even organelles. In contrast, large extracellular vesicles (large EVs) are operationally defined by physical size (diameter >200 nm) and encompass heterogeneous subtypes (e.g., microvesicles, oncosomes, and apoptotic bodies) with biogenesis mechanisms independent of cell migration. Our study demonstrated migrasome-specific functions through three lines of evidence. Negative-staining TEM revealed canonical migrasomes—vesicles with retraction fibers and internal vesicles—in thymic EV isolates (Fig. 3E). In vivo, TSPAN4-GFP-labeled BM-MSC delivered functional migrasomes to thymic tissue, recapitulating the effects on parental cells (Fig. 3H-Q). Critically, both pharmacological (blebbistatin) or genetic (Tspan4KD) suppression of migrasome biogenesis abolished thymic repair (Fig. 6), establishing migrasomes—not other EVs—as the specific therapeutic mediators.

During zebrafish embryonic development, monocytes deposit migrasomes enriched with VEGFA and CXCL12, providing a “’vanguard” for capillary neogenesis and guiding subsequent cell migration and organ morphogenesis [33]. Our findings extend this mechanism to thymic regeneration, demonstrating that migrasomes similarly promote adult tissue repair by driving target cell proliferation. Notably, we identified the Pin1 protein as a novel migrasome cargo with unique significance. As a peptidyl-prolyl isomerase, Pin1 facilitates the G1/S phase transition by modulating conformational changes in cell cycle proteins [41]. This mechanism differs from known growth factor or chemokine signaling (e.g., VEGF/CXCL12), revealing a new paradigm wherein migrasomes directly deliver core cell cycle regulators to trigger target cell proliferation. These findings provide critical insights into the multidimensional functions of migrasomes in tissue regeneration.

In stroke models, angiogenesis and neurogenesis exhibit spatiotemporal coupling. Our data indicate that BM-MSC-derived migrasomes significantly improve neurological function, although the specific effector molecules (e.g., presence of VEGFA) involved require further validation. Post-stroke mitochondrial dysfunction in neurons and immune cells exacerbates secondary injury [44]. In 2021, Yu’s team discovered that migrasomes mediate mitocytosis by expelling damaged mitochondria to maintain intracellular mitochondrial homeostasis [38]. Whether BM-MSC exert neuroprotection via functional mitochondrial supplementation or damaged mitochondrial clearance warrants further investigation. Furthermore, our previous study demonstrated that BM-MSC can transfer mitochondria to neutrophils via migrasomes, thereby promoting mitocytosis in neutrophils and conferring protection in cerebral amyloid angiopathy (CAA) [45]. Moreover, post stroke blood-brain barrier (BBB) disruption post-stroke enables immune cell infiltration and neuroinflammation. Previous studies have shown that macrophage lineage cell-derived migrasomes activate complement-dependent BBB damage [46], and gastric cancer migrasomes carrying the ATF6 protein disrupt BBB integrity [47]. We propose that BM-MSC-derived migrasomes mediate neuroprotection through three pathways: (1) Direct neuroprotection: Delivery of neurotrophic factors or metabolic modulators to injured brain regions; (2) Mitochondrial quality control: Supplementation of functional mitochondria or clearance of damaged mitochondria; (3) Barrier restoration: Re-establishment of BBB integrity to suppress inflammatory infiltration.

Existing evidence confirms that migrasomes play pivotal roles in physiological processes (e.g., embryogenesis and angiogenesis) and pathological states (e.g., tumor metastasis), exhibiting marked context-dependent functionality. Distinct cell types release migrasomes carrying divergent “molecular cargoes” in specific microenvironments, thereby eliciting different biological effects. Migrasome biogenesis involves three stages: nucleation, maturation, and expansion, which are initiated by sphingomyelin synthase 2 (SMS2) and regulated by the PI (4,5) P₂-Rab35 axis [48, 49]. Our proteomics data revealed enrichment of Pin1 and other regulatory proteins in BM-MSC migrasomes, yet the mechanisms governing selective cargo loading remain elusive. Furthermore, whether other proteins within migrasomes contribute to TEC proliferation remains to be explored. Finally, as a cell-free agent, migrasomes circumvent the ethical and safety concerns associated with stem cell transplantation, highlighting their translational potential.

Despite these advancements, several limitations warrant consideration. First, the mechanism by which migrasomes target mTEC remains unclear. Prior evidence suggests that BM-MSC homing relies on the CCL25‒CCR9 axis [50]; further investigation is needed to determine whether TECs express specific receptors for migrasome docking or whether passive diffusion mediates BTB traversal. Elucidating these mechanisms could enhance targeting efficiency. Additionally, in addition to Pin1, migrasomes likely carry additional functional molecules (e.g., mRNAs and mitochondrial fragments). Their contributions to thymic repair and synergy with Pin1 require clarification through multiomics profiling.

This study opens several avenues for future research. First, clinical translation will require scaling migrasome production and optimizing delivery protocols. Standardizing migrasome isolation and storage while ensuring batch-to-batch consistency will be critical for regulatory approval. Second, we aimed to evaluate migrasomes in aging-related thymic involution or chemotherapy-induced immunosuppression models.

## Materials and methods

### Animals

A total of 150 wild-type C57BL/6J mice, comprising 130 males and 20 females, were used in this study. This cohort included 120 young adult mice (2 months old; body weight: 20–30 g), 20 aged male mice (18 months old; body weight: 30–40 g), and 10 neonatal pups (6 days old). Young adult and aged mice were supplied by GemPharmatech Co., Ltd (Nanjing, China; License No. SCXK(Yue)2020-0054). Neonatal mice were obtained from the Guangdong Medical Laboratory Animal Center (Guangzhou, China). All the mice were housed in a specific pathogen-free facility under a 12-hour light/dark cycle with controlled temperature (24 ± 2 °C) and humidity (30–70%). The animals had ad libitum access to food/water and were group-housed (5 per cage). The mice were euthanized on day 14 via isoflurane overdose (RWD; Shenzhen, China; Cat# R510-22). Animal protocols were approved by the Institutional Animal Care and Ethics Committee of Guangdong Academy of Sciences (Approval No. K2024-01–130-480; February 20, 2024) and conducted in compliance with the NIH Guide for the Care and Use of Laboratory Animals (8th ed., 2011) and ARRIVE guidelines (Percie du Sert et al., 2020).

### Isolation of human bone marrow mesenchymal stem cells (BM-MSC)

Heparinized bone marrow aspirates were obtained from healthy donors (recruited via the Third Affiliated Hospital of Sun Yat-sen University) after providing informed consent. BM-MSC were isolated via Ficoll-Paque (1.077 g/mL; GE Healthcare, Cat# 17−1440-02) density gradient centrifugation. The cells were cultured in MSC basal medium (Gibco, Cat# A11577-01) supplemented with BM-MSC growth supplement (Gibco; Cat# A13829-01). For migrasome inhibition, BM-MSC at 70% confluence were treated with blebbistatin (BLEB, 50 µM, 24 h; MCE, Cat# HY-13441). The study was approved by the Ethics Committee of the Third Affiliated Hospital of Sun Yat-sen University (Approval No. 2022-2; September 2, 2022) following ISSCR guidelines (2021).

### Primary culture of thymic epithelial cells (TEC)

The primary culture of thymic epithelial cells was performed as previously described with modifications [51]. Primary cells were characterized by immunofluorescence staining for the epithelial markers CK5 and CK8 (Fig. S13A).

#### Materials

Six 2–5-day-old neonatal mice were used. The complete media used were as follows: DMEM/F12 supplemented with human HDL (100 µg/mL), transferrin (50 µg/mL), insulin (5 µg/mL), hydrocortisone (2.7 × 10⁻⁷ mol/L), EGF (20 ng/mL), sodium selenite (25 ng/mL), and T3 (1 × 10⁻¹⁰ mol/L). Primary digestion: 1.5 mg/mL collagenase IV (Sigma, Cat# C5138) + 500 U DNase I (Sigma, Cat# D5025) in 10 mL of PBS. Passage digestion: 0.25% trypsin + 15 KU DNase I in 10 mL of Ca²⁺/Mg²⁺-free PBS.

#### Primary culture protocol

The neonates were disinfected with 75% ethanol for 30 s and placed in a biosafety cabinet. Thymic glands were excised via midline thoracic incisions and transferred to DMEM/F12. The samples were minced into 1 mm³ fragments and washed twice with complete medium (300 × g, 5 min). The mixture was digested with primary digestion solution at 37 °C for 90 min with gentle agitation every 10 min. The mixture was subsequently centrifuged (300 × g, 5 min) and washed twice with medium. Thirty to forty fragments were plated in a 25 cm² flask with 5 mL of complete medium. Nonadherent debris was removed after 3 days. The epithelial cell islands emerged within 2‒3 days and reached 80% confluency within 2‒3 weeks.

#### Subculture protocol

At confluency, the cells were digested with 2 mL of passage mixture (37 °C, 15–20 min). The digestion was stopped with 200 µL of 1% soybean trypsin inhibitor (Sigma, Cat# T6522). The cells were subsequently centrifuged (300 × g, 5 min) and passaged at a 1:3 ratio in fresh medium.

### Murine models of acute cerebral ischemic stroke

Focal cerebral ischemia was established via the transient middle cerebral artery occlusion (tMCAO) paradigm, which was modified from established protocols [52]. The animals were anesthetized with isoflurane (5% in 1 L/min gas flow for induction, 1.5% for maintenance in 30% O₂/70% N₂O) with the core temperature regulated at 37.0 ± 0.5 °C via a feedback-controlled heating pad. Preemptive analgesia consisted of meloxicam (2.0 mg/kg, s.c.) administered 1 h before incision, combined with local lidocaine infiltration (1.5 mg/kg) at the surgical margins. Postoperatively, meloxicam (0.5 mg/kg, s.c.) was injected daily from 24 to 72 h to manage inflammatory pain. Through microsurgical exposure of the carotid bifurcation, a silicone-coated monofilament (Doccol Corp, 6 − 0) was retrograde introduced into the external carotid stump and advanced 9–11 mm through the internal carotid artery to achieve MCA trunk occlusion. Laser Doppler perfusion imaging (PeriFlux 5000, Perimed AB) confirmed the efficacy of vascular occlusion through real-time cortical blood flow monitoring at bregma − 2 mm coordinates. Regional cerebral blood flow (CBF) was continuously recorded during the preischemia, ischemia, and 15-min postreperfusion phases (see Supplementary Fig. 1C for group-specific data). Animals demonstrating < 70% perfusion reduction from baseline (calculated as ischemic core CBF/preocclusion CBF ×100%) or exhibiting intraoperative mortality were eliminated per predefined exclusion criteria. After 60 min of ischemia, controlled reperfusion was achieved through withdrawal of the filament under antithrombotic surveillance. Sham controls underwent identical cervical dissection without vascular occlusion.

### BM-MSC transplantation and migrasome administration in mice

For transplantation, 2 × 10^6^ BM-MSC (passages 5–8) in 0.2 mL of PBS were infused via the retro-orbital venous sinus 2 h post-tMCAO. For migrasome delivery, BM-MSC-derived migrasomes (10 mg/kg in 0.2 mL PBS) were administered through the same route at the same time point (2 h post-induction). This standardized protocol eliminates temporal variables as confounders in therapeutic efficacy comparisons.

### Assessment of neurological function

Modified Garcia scores (0–3 scale across five domains: body proprioception, vibrissae touch, limb symmetry, lateral turning, and forelimb walking) were evaluated daily for 3 days after tMCAO.

### Infarct volume analysis

Six serial coronal sections obtained at regular intervals (2 mm spacing) through the middle cerebral artery territory were subjected to immunohistochemical labeling using a monoclonal neuronal nuclei marker (NeuN; clone EP3978, dilution 1:1000; Abcam ab177487). Digital image analysis was performed on systematically sampled tissue sections by an independent researcher blinded to the experimental conditions via ImageJ 1.53e software (National Institutes of Health). Structural damage quantification accounts for tissue edema via the following calculation: Final infarct volume (mm³) = (Vcontra - Vipsi_noninfarct) × section thickness × sampling interval, where Vcontra represents the contralesional hemispheric volume and Vipsi_noninfarct indicates preserved neuronal tissue volume in the injured hemisphere.

### Behavioral tests

Sensorimotor functions were assessed via rotarod and foot-fault tests performed 1–3 days before tMCAO and 3–5 days after tMCAO. Cognitive functions were evaluated with the Morris water maze (WTIM) (days 9–13 post-tMCAO) and novel object recognition test (ORTM) (day 11 post-tMCAO). A schematic timeline is provided in Supplementary Fig. 11A.

#### Novel object recognition

The test was conducted in an open-field arena containing two geometrically identical polypropylene objects. After a 60-min familiarization phase with identical objects, one object was replaced with a novel stimulus (matched in size but differing in surface texture) for a 10-min test phase. Exploration time (nose-point proximity ≤ 2 cm) was quantified via automated video tracking (EthoVision 15.0, Noldus). Cognitive performance was assessed by the normalized recognition index (RI = Tnovel/[Tnovel + Tfamiliar] ×100%) and discrimination index (DI = [Tnovel – Tfamiliar]/[Tnovel + Tfamiliar]).

#### Morris water maze test

Hippocampus-dependent spatial learning was evaluated on postoperative days 9–13 via a modified protocol. The apparatus consisted of a circular pool (150 cm diameter) filled with titanium dioxide-opacified water and a hidden acrylic platform (11 × 11 cm) submerged 20 mm below the surface. Hidden platform training (days 9–12): Four daily trials (60 s/trial) were conducted with randomized entry points. The mice were allowed 30 s on the platform after each trial, with spatial cues maintained in the testing room. Probe trial (day 13): The platform was removed, and the time spent in the target quadrant during a 60-sec session was recorded to assess spatial memory retention.

#### Rotarod

Preoperative acclimatization included 3 consecutive days of speed-ramping training (0–300 rpm over 360 s), with day 3 performance used as the baseline. Post tMCAO assessments (day 3) employed identical parameters (6-min acceleration phase followed by constant speed). Trials terminated when an animal disengaged from the rod or underwent ≥ 2 cycles of passive rotation. Motor persistence latency (fall/spin duration) was calculated as the average of three trials.

#### Foot fault

Rodents underwent preoperative gridwalk acclimatization for 3 consecutive days on a 40 × 20 cm elevated grid (4 cm² apertures), with baseline forelimb coordination quantified from day 3 performance metrics. Post-tMCAO assessments on day 3 involved 60-second trials recorded under controlled lighting. Total ambulatory steps and forelimb misplacements (limb descent below the grid plane) were manually quantified by two independent observers blinded to the treatment groups, with the error frequency expressed relative to motion intensity (faults per 100 steps).

### Isolation of BM-MSC-derived migrasomes

BM-MSC cultures at 50% confluence provided conditioned media for vesicle isolation. Sequential differential centrifugation (1,000 g ×10 min →4,000 g ×20 min) was used to clarify the supernatants prior to ultracentrifugation (100,000 g ×70 min, Optima XE-100, Beckman Coulter) for EV enrichment. Migrasome harvesting required fibronectin-coated substrates (0.1 µg/cm², Corning^®^ BioCoat™) with trypsinization (0.125% TrypLE™) at 50% confluence. The processed supernatants were subjected to staged clarification (1,000 × g/4,000 × g), followed by pelleting at 20,000 × g for 30 min. Contaminant elimination protocols involve iterative washing cycles (PBS + matching centrifugal forces).

### Bulk RNA sequencing and data analysis

Thymic RNA was isolated via TRIzol-based protocols with RNA integrity validation (RIN ≥ 8.0, Bioanalyzer 2100). Sequencing libraries were prepared via the NEBNext Ultra II Directional RNA Library Kit (E7760S) following ribosomal RNA depletion (NEBNext rRNA Removal Kit). Pooled libraries were subjected to 150 bp paired-end sequencing on NovaSeq 6000 SP flow cells (Illumina, 40 M reads/sample). Differentially expressed genes were identified via DESeq2 (v1.40.2) with significance thresholds (FDR-adjusted *p* < 0.05, |log2FC|≥1). Functional annotation was performed through clusterProfiler (v4.4.4) via the current GO (2023–10 release) and Reactome (v84) databases.

Quality control of the bulk RNA-seq data was performed through principal component analysis (PCA), relative log expression (RLE) diagnostics, and expression distribution assessment (Fig. S14). PCA was conducted on the basis of the FPKM-normalized gene expression values across all the samples. The raw count matrices were processed via edgeR. Library size normalization was performed via calcNormFactors with TMM weighting, followed by calculation of variance-stabilized log₂ (CPM + 1) values. The RLE diagnostics were visualized via the EDASeq package. Expression distributions were evaluated through: density plots and boxplots of log₂-transformed raw counts (log₂ (count + 1)) to mitigate variance inflation from highly expressed genes. Analogous visualizations of log₂ (FPKM + 1) values to account for gene length and sequencing depth effects. This comprehensive suite of quality assessment analyses (PCA, RLE, expression distribution diagnostics based on the basis of counts and FPKM) demonstrates that our bulk RNA-seq data from thymic tissue are of high quality and free from major technical artifacts.

### ScRNA-seq and data analysis

Thymic cell suspensions were prepared from a total of 12 mice (*n* = 4 biologically independent mice per group for Veh, BM-MSC, and Migrasome treatments). Cell suspensions were centrifuged at 800 × g for 5 min. Pelleted cells underwent CD45^−^ based enrichment via anti-mouse CD45 MicroBeads and magnetic-activated cell sorting (MACS). To ensure sufficient cell numbers for capturing rare TEC subsets and to minimize inter-individual variation, CD45^−^ enriched cells from the 4 mice within the same treatment group were pooled at equal quantities, resulting in one final sample pool per group for sequencing. The cells were sorted at a CD45⁻:CD45⁺ ratio of 5:1 to further enrich TEC.

Single-cell libraries were prepared with sorted cells (viability > 85%) loaded onto chromium microfluidic chips (10X Genomics) employing 3’ chemistry. All samples from the three groups were processed simultaneously in a single batch for tissue processing, cell sorting, library construction, and sequencing to prevent technical batch effects. Barcoding was performed via the Chromium Controller (10X Genomics). Reverse transcription and library construction were performed via the Chromium Single Cell 3’ v2 Reagent Kit (10X Genomics) per the manufacturer’s protocol. Libraries were sequenced on Illumina platforms (paired-end).

The raw sequencing data were subjected to quality control via fastp with adapter trimming and quality filtering (Q20 threshold). Processed reads were demultiplexed and aligned to the mm10 reference genome using Cell Ranger with default parameters. Digital gene expression matrices were generated through unique molecular identifier (UMI) counting.

Downstream analysis was performed in Seurat (v5.0) with sequential filtering: genes detected in < 3 cells were excluded, cells with < 1000 expressed genes were excluded and potential doublets identified by scDblFinder were removed. The cell clusters were annotated based on canonical markers. Differentially expressed genes (adjusted *p* < 0.05, |log₂FC|>1) underwent functional enrichment via clusterProfiler with gene length bias correction. Significantly enriched GO terms and KEGG pathways (FDR < 0.05) were reported. Pseudotime trajectories were reconstructed using *monocle3* with default graph learning parameters.

### Liquid chromatography-tandem mass spectrometry (LC‒MS/MS) analysis

The migrasome protein composition was characterized via liquid chromatography tandem mass spectrometry (LC‒MS/MS). Proteins were resolved via SDS‒PAGE (12% separating gel) and stained with Coomassie Brilliant Blue R-250. Gel slices containing target proteins were subjected to in-gel tryptic digestion (16 h, 37 °C) prior to analysis. Mass spectrometry was performed via a Q Exactive system (Thermo Fisher Scientific) with nanoelectrospray ionization (NanoFlex source). Raw spectral data processing, including protein identification and quantitative analysis, was executed through the PEAKS Studio platform (v8.5) under strict filtering criteria (FDR < 1%).

### Histological analyses

Thymus samples were fixed in 4% paraformaldehyde (PBS, pH 7.4) for 48 h, followed by paraffin embedding. Next, 8-µm-thick sections were subjected to xylene-mediated deparaffinization and rehydration in graded ethanol. The tissue architecture was visualized through Mayer’s hematoxylin (6 min)/eosin Y (90 s) staining, with optical microscopy (Nikon Eclipse Ci-L) imaging at 40–400× magnification for cortical–medullary demarcation analysis.

### Flow cytometric analysis

Flow cytometry was performed using a BD Biosciences FACS analyzer. The data were analyzed with FlowJo software (v10.8), and the gating strategies are detailed in Supplementary Fig. 3.

#### Sample preparation

Peripheral blood was collected in heparinized tubes, lysed with ACK buffer (Gibco, Cat# A1049201), and washed with PBS. Brain tissue: Ipsilateral hemispheres were digested with 0.25% trypsin-EDTA (37 °C, 25 min) and filtered through 70-µm strainers. Thymic epithelial cells were mechanically dissociated, digested with collagenase (37 °C, 30 min), and filtered through 70-µm strainers. Thymus/spleen immune cells: Mechanically homogenized and filtered through 70-µm strainers.

#### Staining protocol

Single-cell suspensions were fixed/permeabilized via the Intracellular Fixation & Permeabilization Buffer Set (Invitrogen, Cat# 00−5123-43, 00–5223-56). The following antibodies were used: PerCP-anti-mouse CD45 (1:400; Biolegend 103132, clone 30-F11), FITC-anti-mouse CD3 (1:400; Biolegend 100204, clone 17A2), PE-anti-mouse CD19 (1:400; Biolegend 152408, clone 1D3), PE/Cy7-anti-CD11b (1:400; Biolegend 101216, clone M1/70), BV421-anti-F4/80 (1:400; Biolegend 123132, clone 8M8), APC/Cy7-anti-Ly6G (1:400; Biolegend 108424, clone RB8-8C5), FITC-anti-Ki67 (1:200; BD 556026, clone B56), APC-anti-EPCAM (1:400; Biolegend 118213, clone G8.8), PE/Cy7-anti-Ly51 (1:400; Biolegend 108313, clone M5/114.15.2), anti-AIRE (1:400; Abcam ab243169, clone 5H12), and APC-anti-MHCⅡ(1:400; Biolegend 107607), FITC-anti-CD90 (1:400; Biolegend 328108, clone 5E10), APC-anti-CD34 (1:400; Biolegend 343509, clone 581), PE-anti-CD73 (1:400; Biolegend 344003, clone AD2), PE-anti-CD105 (1:400; Biolegend 120414, clone MJ7/18), APC-anti-mouse IL-2 (Biolegend, 503809), PE/Cyanine7-anti-mouse IFN-γ (Biolegend, 505825), APC/Cy7-streptavidin (Biolegend 405208), Ulex europaeus agglutinin-1 (UEA-1; Vector Labs B-1065).

### Immunofluorescence staining

For in vivo experiments, the mice were euthanized at 7 or 14 days post-tMCAO and transcardially perfused with 10 mL of PBS followed by 10 mL of 4% paraformaldehyde (PFA, pH 7.4). The brains were sectioned coronally (25 μm thick), while the thymus tissues were embedded in optimal cutting temperature (OCT) compound and cryosectioned at 5 μm. In vitro experiments, TECs or BM-MSC were seeded on poly-L-lysine (Sigma, Cat# P2636)-coated coverslips. The cells were fixed with 4% PFA for 20 min at room temperature. Sections or fixed cells were permeabilized/blocked with PBS containing 0.03% Triton X-100 and 3% BSA for 1 h at room temperature. The sections were incubated with the following antibodies overnight at 4 °C: rabbit anti-NeuN (1:300; Abcam ab177487), mouse anti-MBP (1:300; Merck Millipore MABT1499), rabbit anti-EPCAM (1:1000; Servicebio GB11274), rabbit anti-CK5 (1:1000; Servicebio GB111246), mouse anti-CK8 (1:1000; Servicebio GB12233), mouse anti-Ki67 (1:500; Abcam ab279653), rabbit anti-P21 (1:500; Affinity Biosciences AF6290), and rabbit anti-Pin1 (1:500; Abcam ab192036). The washed samples were incubated for 1 h at room temperature (light-protected): Cy3-conjugated anti-rabbit (1:1000; Jackson 115–165–003), Alexa Fluor 488-conjugated anti-mouse (1:1000; Jackson 112–545–003), and Alexa Fluor 647-conjugated anti-rabbit (1:1000; Jackson 111–605–003) antibodies. The cell membranes were labeled with wheat germ agglutinin (WGA, 1 µg/mL; Invitrogen W7024).

### Lentiviral infection of BM-MSC

#### TSPAN4 overexpression

To overexpress *Tspan4* in BM-MSC, human TSPAN4 cDNA was cloned and inserted into the lentiviral transfer vector TK-PCDH-copGFP-T2A-Puro via NheI/BamHI restriction sites, whose sequence was as follows: gctagcATGGCTAGGGCATGCAGGCTGTCAAGTACCTGATGTTCGCCTTCAACCTGCTCTTCTGGCTGGGCGGATGTGGTGTGCTGGGCGTGGGCATCTGGCTGGCTGCCACCCAGGGCTCTTTCGCCACACTCAGCTCTAGCTTTCCAAGCCTTTCTGCCGCTAACCTGCTGATCATCACTGGTGCTTTCGTGATGGCAATCGGCTTCGTCGGCTGTCTGGGCGCTATCAAAGAGAACAAGTGCCTCTTGCTGACATTCTTTCTCTTGCTGCTGCTGGTGTTTCTGCTGGAGGCTACAATCGCCATCTTGTTCTTCGCCTATACCGACAAGATTGACAGGTACGCACAGCAGGACCTGAAGAAAGGCCTGCATCTGTATGGCACACAGGGTAACGTGGGCTTGACCAACGCCTGGTCTATCATTCAGACAGACTTCAGATGCTGCGGCGTGAGCAACTACACAGACTGGTTTGAGGTCTACAACGCTACCAGAGTGCCTGACAGCTGCTGCTGCTTGGAGTTTAGCGAATCTTGTGGACTGCATGCA.

#### TSPAN4 knockdown

A *Tspan4*-targeting shRNA was designed. From the three candidate sequences, the most efficient shRNA target sequence: GATCGTGGATAGCTACGACGTGATTCCTCGAGGAATCACGTCGTAGCTATCCATTTTTT was cloned and inserted into the lentiviral transfer vector pLVX-shRNA2-ZsGreen1-Puro via BamHI/EcoRI.

#### Pin1 overexpression

To overexpress *Pin1* in BM-MSC, human *Pin1* cDNA was cloned and inserted into the lentiviral transfer vector pLV-PGK-ZsGreen(GSP2A)PURO-CMV via XhoI-EcoRI restriction sites, whose sequence was as follows: CTCGAGGCCACCATGGCGGACGAGGAGAAGCTGCCGCCCGGCTGGGAGAAGCGCATGAGCCGCAGCTCAGGCCGAGTGTACTACTTCAACCACATCACTAACGCCAGCCAGTGGGAGCGGCCCAGCGGCAACAGCAGCAGTGGTGGCAAAAACGGGCAGGGGGAGCCTGCCAGGGTCCGCTGCTCGCACCTGCTGGTGAAGCACAGCCAGTCACGGCGGCCCTCGTCCTGGCGGCAGGAGAAGATCACCCGGACCAAGGAGGAGGCCCTGGAGCTGATCAACGGCTACATCCAGAAGATCAAGTCGGGAGAGGAGGACTTTGAGTCTCTGGCCTCACAGTTCAGCGACTGCAGCTCAGCCAAGGCCAGGGGAGACCTGGGTGCCTTCAGCAGAGGTCAGATGCAGAAGCCATTTGAAGACGCCTCGTTTGCGCTGCGGACGGGGGAGATGAGCGGGCCCGTGTTCACGGATTCCGGCATCCACATCATCCTCCGCACTGAGTGAGAATTC.

#### Pin1 knockdown

A *Pin1*-targeting shRNA was designed. From the four candidate sequences, the most efficient shRNA target sequence: GATCGGCCATTTGAAGACGCCTCGTTCTCGAGAACGAGGCGTCTTCAAATGGCTTTTTT was cloned and inserted into the lentiviral transfer vector pLVX-shRNA2-Puro via BamHI/EcoRI.

#### Lentivirus production and transduction

The recombinant plasmid was amplified from DH5α *E. coli* and purified via the NucleoBond Xtra Midi EF Kit (Macherey-Nagel, Cat# 740420). The transfer vector, packaging plasmid pSPAX2 (Addgene #12260), and envelope plasmid pMD2. G (Addgene #12259) were mixed at a 3:2:1 mass ratio in OPTI-MEM (Gibco, Cat# 31985070). PEI MAX transfection reagent (Polysciences, Cat# 23966) was added at a DNA: PEI ratio of 1:1. After 12 h, the medium was replaced with complete DMEM. The viral supernatant was harvested at 48 h post transfection and clarified by centrifugation (800 × g, 10 min). BM-MSC (passage 5) were transduced with viral supernatant (MOI = 20) in the presence of polybrene via centrifugation (1000 × g, 32 °C, 2 h), followed by 48 h of culture in fresh medium. The transduction efficiency was validated via RT‒PCR (primers in Table S1) and Western blotting with anti-rabbit-TSPAN4 (Signalway Antibody, ab109264, 1:1000) and anti-rabbit-Pin1(Abcam, ab192036, 1:1000) antibodies.

### Western blot

Protein was extracted with RIPA buffer (Beyotime, Cat# P0013) and centrifuged (12,000 × g, 15 min, 4 °C). The protein concentration was determined via a BCA assay (Beyotime, Cat# P0012), with 40 µg of total protein loaded per lane. The samples were denatured (95 °C, 5 min) and separated on 12% SDS‒PAGE gels (80 V for 30 min, then 120 V for 60 min). Proteins were transferred to PVDF membranes (Merck Millipore, Cat# ISEQ00010) via wet transfer (100 V, 90 min, 4 °C). The membranes were blocked with 5% BSA/TBST for 1 h at RT. The following primary antibodies were used overnight at 4 °C: mouse anti-Ki67 (1:1,000; Abcam ab279653), rabbit anti-NAMPT (1:1,000; Affinity Biosciences DF6059), rabbit anti-P21 (1:1,000; Affinity Biosciences AF6290), rabbit anti-γH2A. X (1:1,000; Affinity Biosciences AF3187), rabbit anti-TSPAN4 (1:1,000; Signalway Antibody ab109264), rabbit anti-Pin1 (Abcam, ab192036, 1:1000), mouse anti-β actin (Servicebio, GB15001, 1:2000) and mouse anti-GAPDH (1:10,000; Proteintech 60004-1-Ig) antibodies. HRP-conjugated anti-mouse/rabbit IgG (1:5,000; Proteintech SA00001-1/SA00001-2) was added for 1 h at RT. The signals were developed with SuperSignal™ West Pico substrate (Thermo Fisher, 34580) and quantified via Image Lab 6.1 (Bio-Rad).

### Real-time polymerase chain reaction (RT‒PCR) and TREC analysis

Total RNA was isolated via the EScience RNA Extraction Kit (Cat# RN001) according to the manufacturer’s instructions. The RNA purity was verified via a Nanodrop (A260/A280 = 1.8–2.2). One microgram of RNA was reverse transcribed in a 20 µL volume via the EScience Fast Reverse Transcription Kit (Cat# RT001), which contained 1 µL of cDNA, SYBR Green qPCR mix (Dongsheng Biotech, Cat# P2092a), and gene-specific primers (sequences in Supplementary Table S1) in a final volume of 20 µL. Amplification was performed on a QuantStudio 5 Real-Time PCR System (Applied Biosystems) under the following conditions: 95 °C for 30 s (initial denaturation); 40 cycles of 95 °C for 5 s (denaturation) and 60 °C for 34 s (annealing/extension); and melt curve analysis at 95 °C for 15 s, 60 °C for 60 s, and 95 °C for 15 s. The ΔΔCt method was employed with GAPDH as the endogenous reference, with the data normalized to the means of the control groups. For TREC quantification, genomic DNA was isolated from peripheral blood. TREC signal joints were quantified by qPCR using primers specific for the δRec-ψJα recombination event in C57BL/6J mice, with amplification of the T-cell receptor alpha constant region (TRAC) used the endogenous normalization standard (sequences in Supplementary Table S1). The ΔΔCt method was employed with TRAC as the endogenous reference, with the data normalized to the means of the control groups.

### Negative staining transmission electron microscopy (TEM)

Thymus samples were fixed in 4% paraformaldehyde (PBS, pH 7.4) for 48 h, followed by paraffin embedding. Next, 8-µm-thick sections were subjected to xylene-mediated deparaffinization and rehydration in graded ethanol. The tissue architecture was visualized through Mayer’s hematoxylin (6 min)/eosin Y (90 s) staining, with optical microscopy (Nikon Eclipse Ci-L) imaging at 40–400× magnification for cortical–medullary demarcation analysis.

### Enzyme-linked immunosorbent assay (ELISA)

Plasma was isolated from peripheral blood by centrifugation (15,000 × g, 15 min, 4 °C) and stored at −80 °C until analysis. Plasma levels of LBP, LPS, CRP, thymosin α1 (Tα1), thymosin β4 (Tβ4), and thymosin β10 (Tβ10) were measured via the following ELISA kits according to the manufacturers’ protocols: a mouse LBP ELISA Kit (MEIMIAN, Cat# MM-44515M1), a mouse LPS ELISA Kit (MEIMIAN, Cat# MM-0634M1), a mouse CRP ELISA Kit (MEIMIAN, Cat# MM-0074M1), a mouse Tα1 ELISA Kit (MEIMIAN, Cat# MM-44450M1), a mouse Tβ4 ELISA Kit (MEIMIAN, Cat# MM-64220H1), and a mouse Tβ10 ELISA Kit (MEIMIAN, Cat# MM-64224H1).

### Statistical analysis

All the results are presented as the standard deviations (SDs). Prior to inferential testing, all datasets underwent dual diagnostic verification: normality assessment with the Shapiro-Wilk test (significance threshold α = 0.05) and homoscedasticity evaluation with Levene’s test (threshold *P* > 0.10 for variance homogeneity). Parametric tests (independent Student’s *t*-tests or one-way ANOVA) were applied strictly when both assumptions were satisfied. For data violating parametric assumptions (non-normality or heteroscedasticity), Wilcoxon rank-sum tests were systematically implemented for median comparisons between independent samples based on rank-transformed data. All post hoc pairwise comparisons following ANOVA were subjected to Benjamini-Hochberg false discovery rate (FDR) correction. To ensure unambiguous interpretation of FDR-adjusted *q-values* distinct from conventional *p*-values, a dedicated annotation system was adopted: #*q* < 0.05, ##*q* < 0.01, ###*q* < 0.001 (replacing asterisk-based notation). All the statistical tests were two-sided, with α = 0.05 defining significance. Analyses were performed via SPSS Statistics 25.0 package (Nonparametric Tests module v3.1).

## Supplementary Information

Supplementary Material 1.

Supplementary Material 2.

## Data Availability

All data needed to evaluate the conclusions in the paper are presented in the paper and/or the Supplementary Materials. Additional data related to this paper may be requested from the authors. The raw sequence data from the scRNA-seq and RNA-seq datasets have been deposited in the Gene Expression Omnibus (GEO) datasets (accession no. GSE305141).
