# Supplementary figures and images for: BM-MSC-derived migrasomes reverse stroke-induced thymic atrophy and immunosuppression via Pin1 delivery to thymic epithelial cells
Source: J Neuroinflammation. 2025 Nov 15;22:271. doi: 10.1186/s12974-025-03604-2 (PMC12619471; doi:10.1186/s12974-025-03604-2)

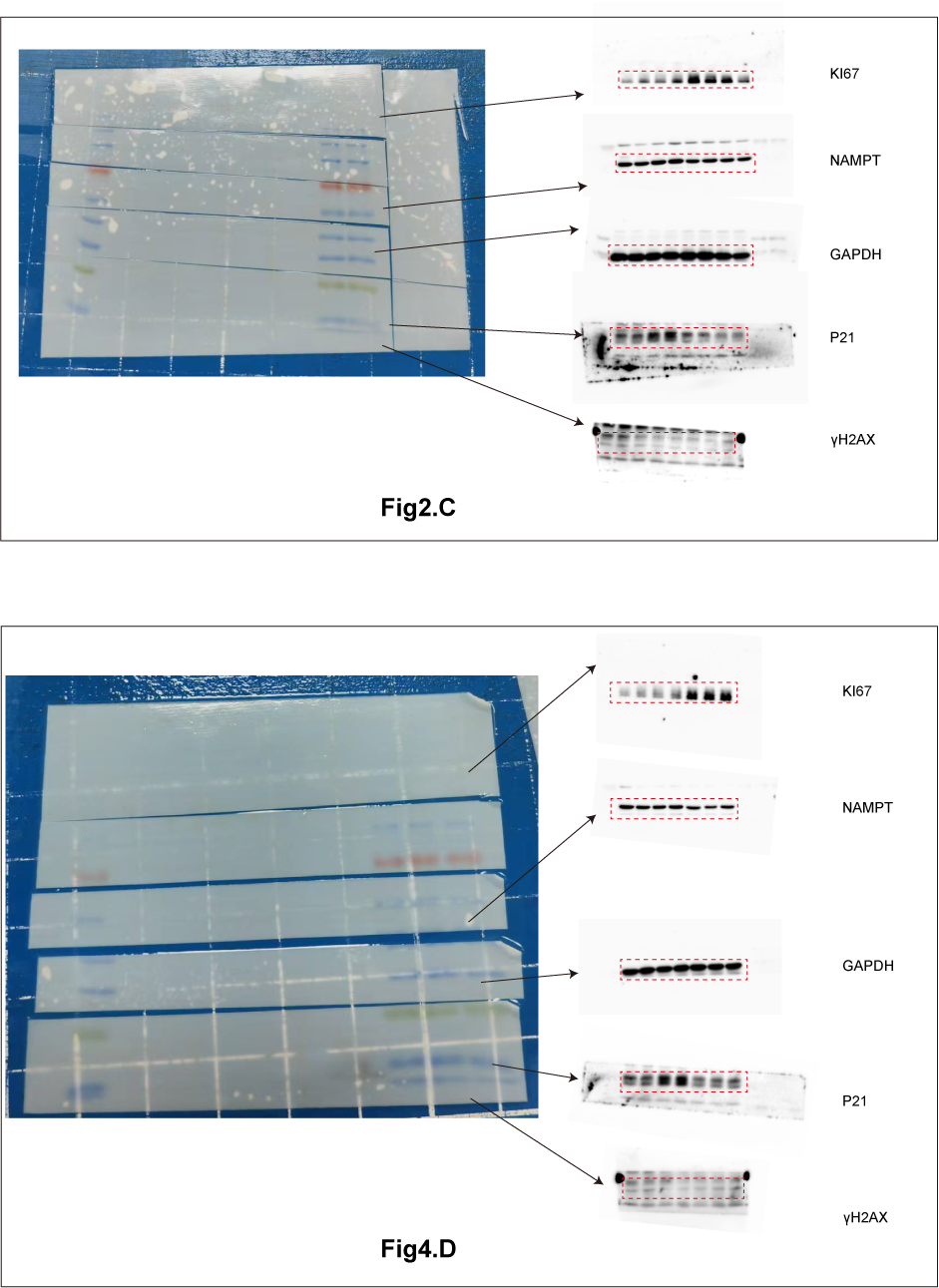


**
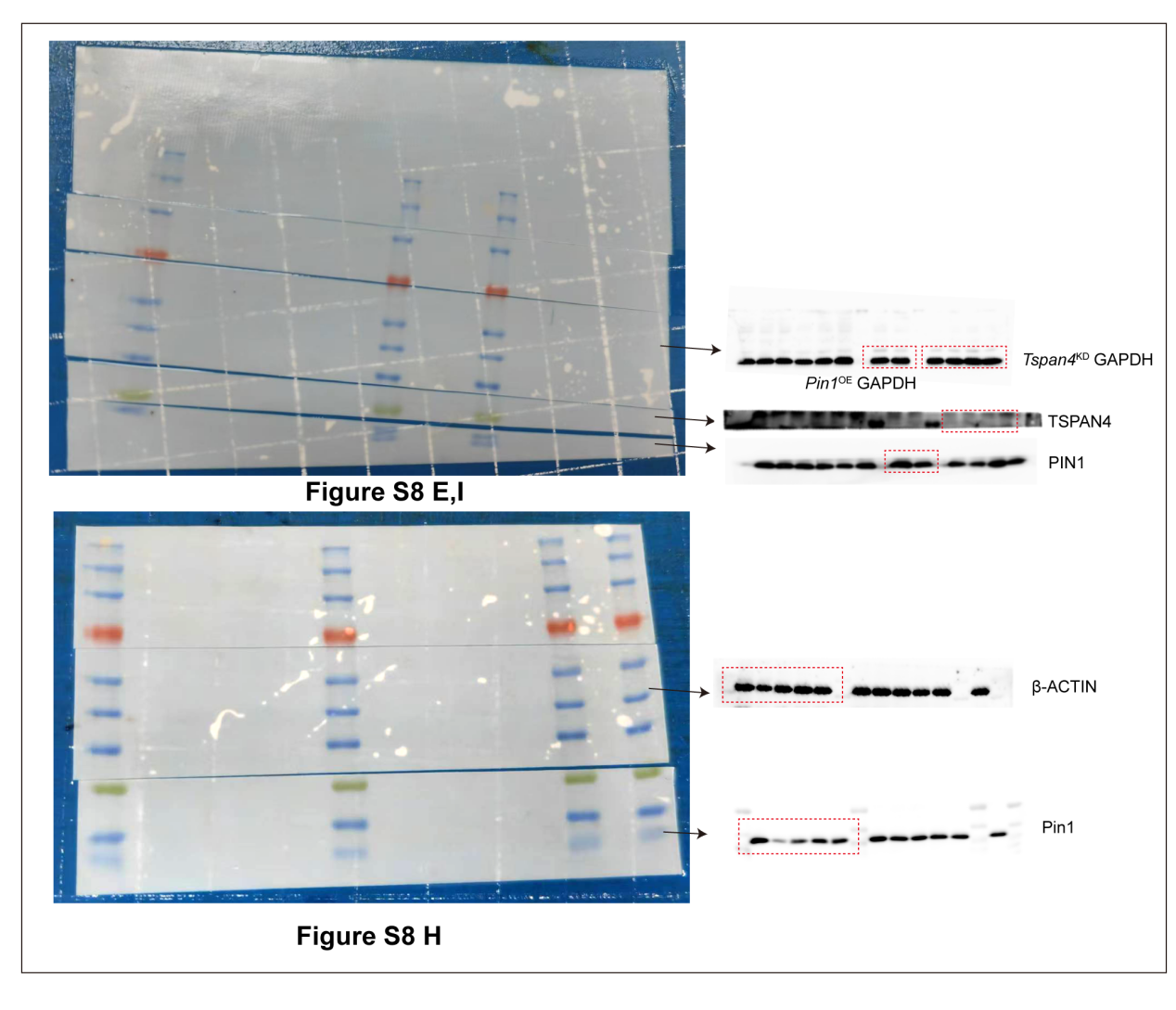
**

**Original protein bands for WB experiments. Related to Fig. 2, Fig. 4 and Fig. S8.**

Supplement: Supplementary file 1 — Supplementary Material 1. [file 12974_2025_3604_MOESM1_ESM.docx]
