# Supplementary material for: BM-MSC-derived migrasomes reverse stroke-induced thymic atrophy and immunosuppression via Pin1 delivery to thymic epithelial cells
Source: J Neuroinflammation. 2025 Nov 15;22:271. doi: 10.1186/s12974-025-03604-2 (PMC12619471; doi:10.1186/s12974-025-03604-2)
Supplement: Supplementary file 2 — Supplementary Material 2. [file 12974_2025_3604_MOESM2_ESM.docx]

Supporting Information

**BM-MSC-Derived Migrasomes Reverse Stroke-Induced Thymic Atrophy and Immunosuppression via Pin1 Delivery to Thymic Epithelial Cells**

*Haotong Yi^1, #^, Mengyan Hu^1, 2, #^, Liling Yuan^1^, Xiaotao Su^1^, Shilin Wu^1^, Tiemei Li^1^, Shisi Wang^1^, Xinmei Kang^1^, Yuxin Liu^1^, Zhiruo Liu^1^, Qin Qin^1^, Weihua yu^3^, Yifan Li^1^, Wei Qiu^1,2 *^, Wei Cai^1,2 *^, Zhengqi Lu^1, *^*

**This file includes:**

**Supplementary Figures**

Figure S1. Characterization of BM-MSCs and Comprehensive Evaluation of Stroke Models.

Figure S2. Intracranial Immune Cell Infiltration in tMCAO Mice.

Figure S3. Flow Cytometry Gating Strategies for Thymic, Splenic, Peripheral Blood, and Brain Immune Cells.

Figure S4. Dynamic changes in peripheral immune profiles during post-stroke immunosuppression.

Figure S5. Peripheral Immune Evaluation in Young Mice.

Figure S6. Peripheral Immune Evaluation in Aged Mice.

Figure S7. Functional assessment of thymic output and T-cell competence.

Figure S8. Validation of engineered BM-MSC models for migrasome research.

Figure S9. Flow Cytometric Tracking of GFP Signal in Thymus of tMCAO Mice.

Figure S10. BM-MSC-derived migrasomes promote thymic regeneration and systemic immune restoration in female mice after ischemic stroke.

Figure S11. Behavioral Assessments in tMCAO Mice.

Figure S12. Comprehensive Analysis of Non-epithelial thymic Populations in scRNA-seq Data.

Figure S13. In Vitro Isolation of TECs and Evaluation of Migrasomes from Bleb-Treated BM-MSCs.

Figure S14. Quality Control of bulk RNA-seq Data.

Figure S15. Flow Cytometry Analysis of Thymic Epithelial Cells and T-Cell Subsets in Migrasome-Treated tMCAO Mice.

**Supplementary Tables:**

Table S1. Primers used in the study.


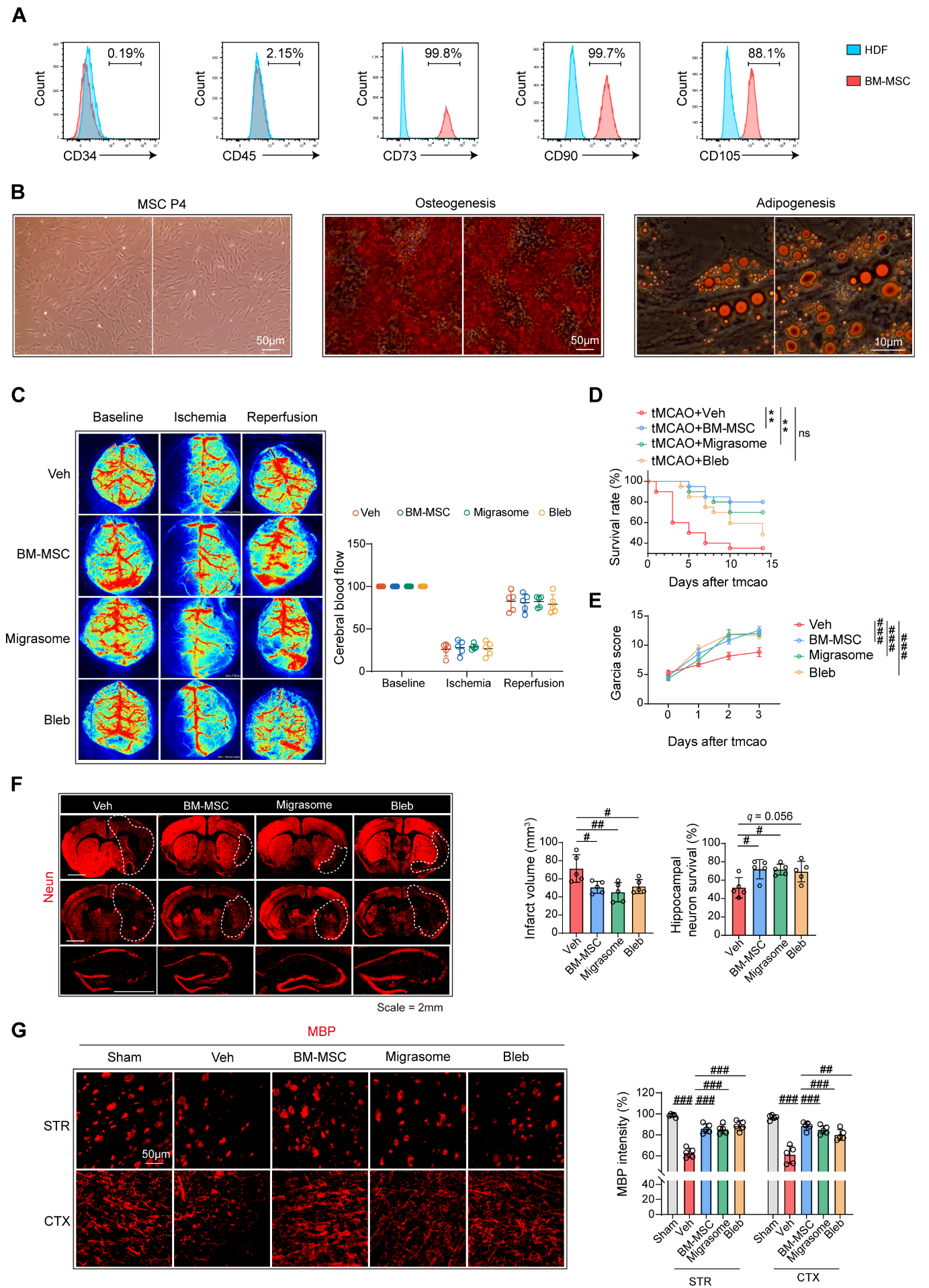


**Figure S1. Characterization of BM-MSCs and Comprehensive Evaluation of Stroke Models.**

**(A)** Purity and stemness marker analysis of BM-MSC (CD34^-^CD45^-^CD73^+^CD90^+^CD105^+^). Experiments repeated three times. **(B)** Representative bright-field images of osteogenic differentiation (Alizarin Red S) and adipogenic differentiation (Oil Red O) in BM-MSCs (P4). Experiments repeated three times. **(C-G)** Male wild-type C57/Bl6 mice underwent 60-minute tMCAO followed by intravenous administration of BM-MSC (2×10⁶ cells/mouse), migrasomes (10 mg/kg, isolated from TSPAN4-GFP-overexpressing BM-MSCs), blebbistatin (Bleb)-pretreated BM-MSC (2×10⁶ cells/mouse), or vehicle (Veh) 2 hours post-reperfusion. **(C)** Regional cerebral blood flow (CBF) at baseline, during ischemia, and 15 minutes post-reperfusion showed no differences between groups (*n*=5 per group). **(D)** Survival curves from 0 to 14 days post-tMCAO (*n*=20 per group). ***p* < 0.01 (Veh vs. BM-MSC, Migrasome and Bleb group), by log-rank test. **(E)** Neurological deficit scores from 0 to 3 days post-tMCAO (Veh *n*=8, BM-MSC *n*=9, migrasome *n*=10, Bleb *n*=8). FDR-corrected *p*-values (*q-values)* were calculated, ###*q* < 0.001, two-way ANOVA (mean ± SD). (**F)** NeuN immunostaining (red) at 14 days post-tMCAO to assess neuronal loss. Dashed lines outline infarct areas. Bar graphs show infarct volume quantification (*n*=5 per group). FDR-corrected *p*-values (*q-values)* were calculated, #*q* < 0.05, ##*q* < 0.01, one-way ANOVA (mean ± SD). **(G)** Representative MBP immunostaining in striatum (STR) and cortex (CTX). Bar graphs show mean fluorescence intensity (MFI) quantification (*n*=5 per group). FDR-corrected *p*-values (*q-values)* were calculated, ##*q* < 0.01, ###*q* < 0.001, one-way ANOVA (mean ± SD).


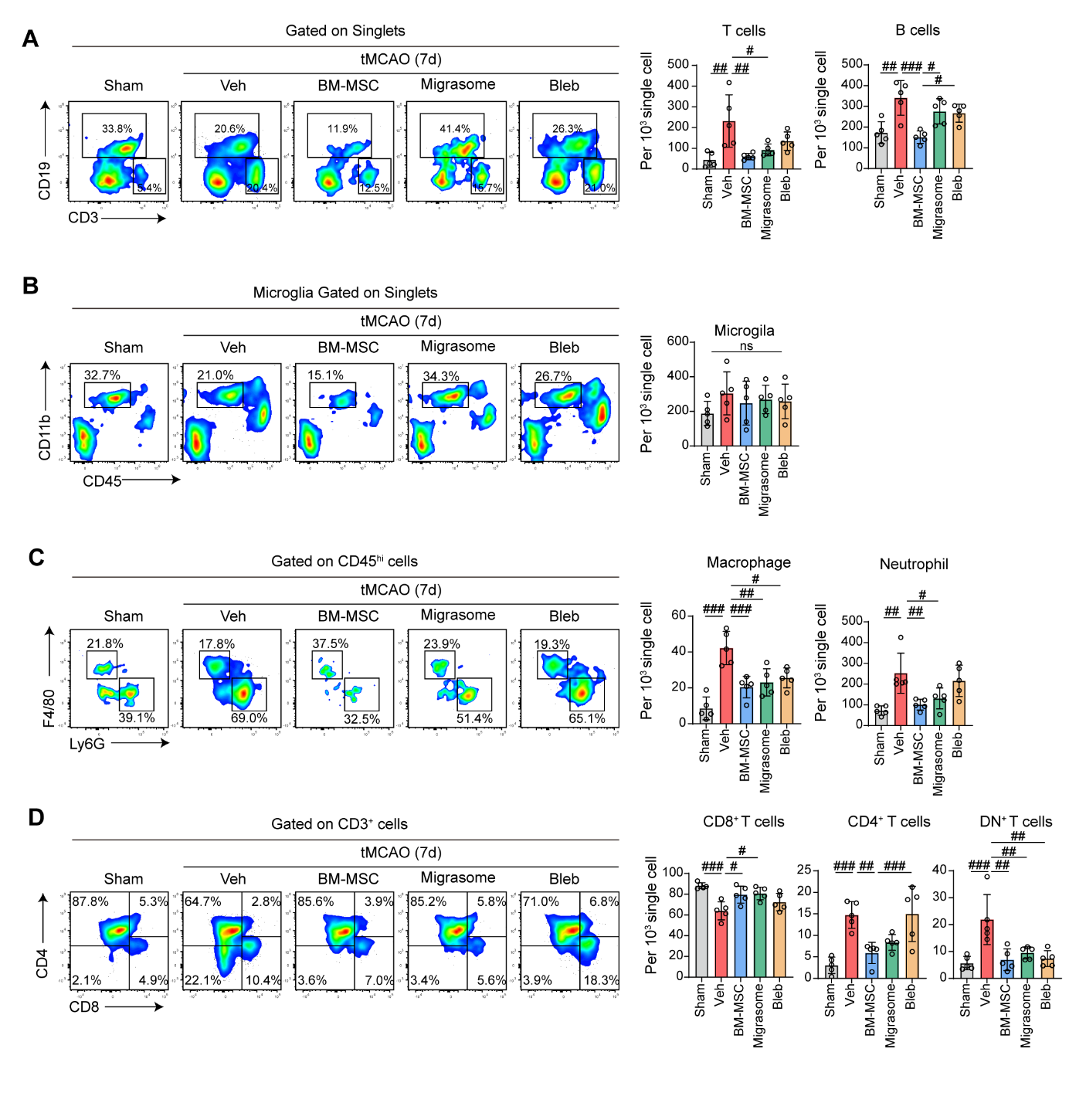


**Figure S2. Intracranial Immune Cell Infiltration in tMCAO Mice.**

Male wild-type C57/Bl6 mice underwent 60-minute tMCAO followed by intravenous administration of BM-MSCs (2×10⁶ cells/mouse), migrasomes (10 mg/kg, isolated from TSPAN4-GFP-overexpressing BM-MSC), blebbistatin (Bleb)-pretreated BM-MSC (2×10⁶ cells/mouse), or vehicle (Veh) 2 hours post-reperfusion. **(A-D)** Flow cytometry analysis of intracranial immune cell infiltration (*n*=5 per group; gating strategies shown in Supplementary Figure 3). **(A)** Proportions of T cells (CD3^+^) and B cells (CD19^+^). **(B)** Proportions of microglia (CD11b^+^CD45^low^). **(C)** Proportions of macrophages (CD11b^+^CD45^hi^F4/80^+^) and neutrophils (CD11b^+^CD45^hi^Ly6G^+^). **(D)** Proportions of T-cell subsets (CD4^+^, CD8^+^, DN, DP). FDR-corrected *p*-values (*q-values)* were calculated, #*q* < 0.05, ##*q* < 0.01, ###*q* < 0.001, one-way ANOVA (mean ± SD).


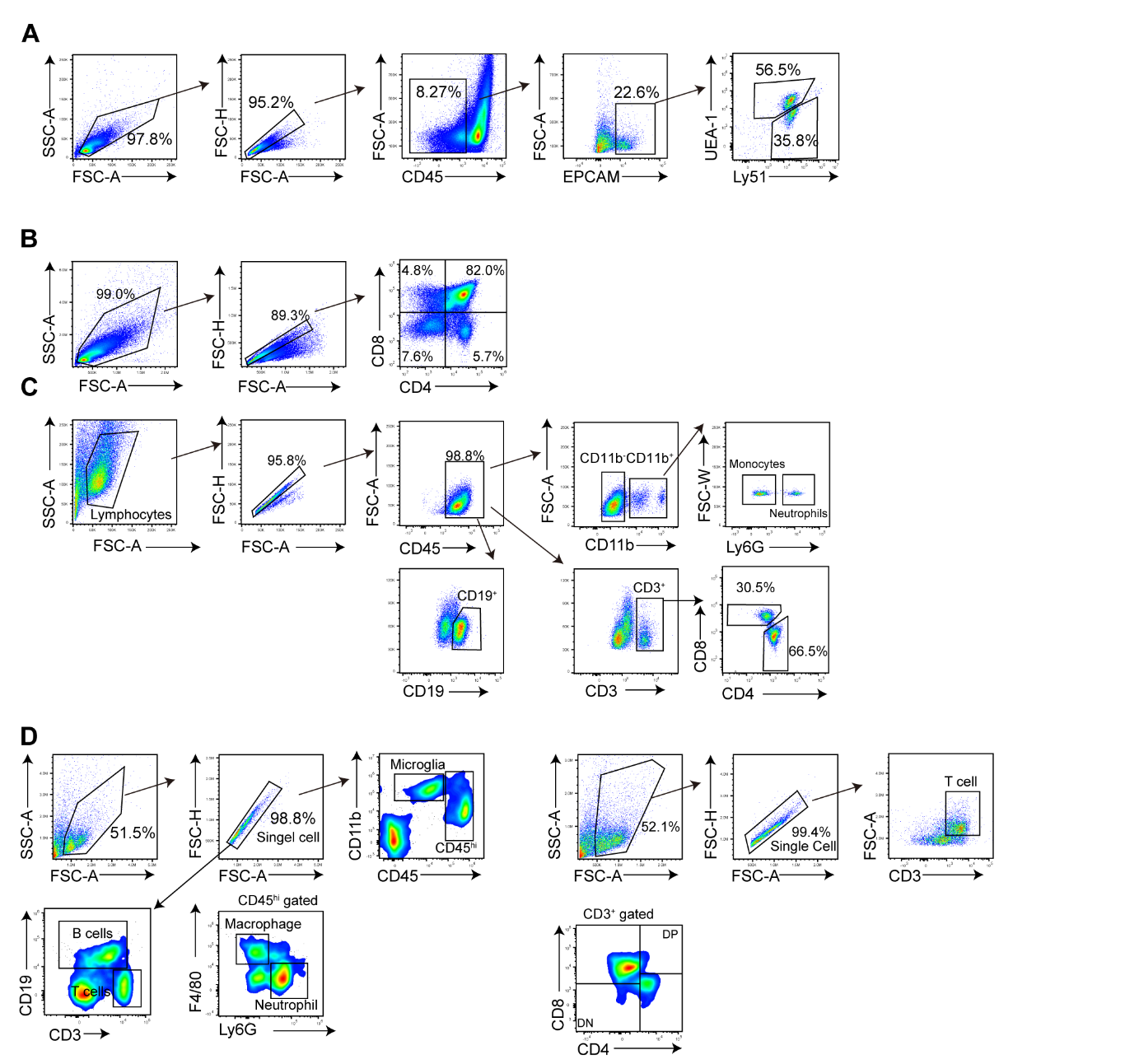


**Figure S3. Flow Cytometry Gating Strategies for Thymic, Splenic, Peripheral Blood, and Brain Immune Cells.**

Representative gating strategies used for flow cytometry analysis: **(A)** Thymic epithelial cells (TEC): Gating for mTEC (CD45^−^EPCAM^+^UEA-1^+^Ly51^−^) and cTEC (CD45^−^EPCAM^+^UEA-1^−^Ly51^+^) (applied to Figure 1G-H, Figure 3P, Figure 6F, and Supplementary Figure 15). **(B)** Thymic T-cell subsets: Gating for double-negative (DN, CD4−CD8−), double-positive (DP, CD4^+^CD8^+^), CD4^+^, and CD8^+^ T cells (applied to Figure 1K, Figure3Q, Figure 6G, and Supplementary Figure 10, 15). **(C)** Splenic and peripheral blood immune cells: T cells (CD45^+^CD3^+^), B cells (CD45^+^CD19^+^), CD8^+^ T cells (CD45^+^CD3^+^CD8^+^CD4^-^), CD4^+^ T cells (CD45^+^CD3^+^CD8^−^CD4^+^), macrophages (CD45^+^CD11b^+^Ly6G^−^), neutrophils (CD45^+^CD11b^+^Ly6G^+^) (applied to Supplementary Figure 4-6, 10). **(D)** Brain-infiltrating immune cells: B cells (CD19^+^), T cells (CD3^+^), Macrophages (CD45^hi^F4/80^+^Ly6G^-^), neutrophils (CD45^hi^F4/80^-^Ly6G^+^), CD8^+^ T cells (CD3^+^CD8^+^), CD4^+^ T cells (CD3^+^CD4^+^) (applied to Supplementary Figure 2).


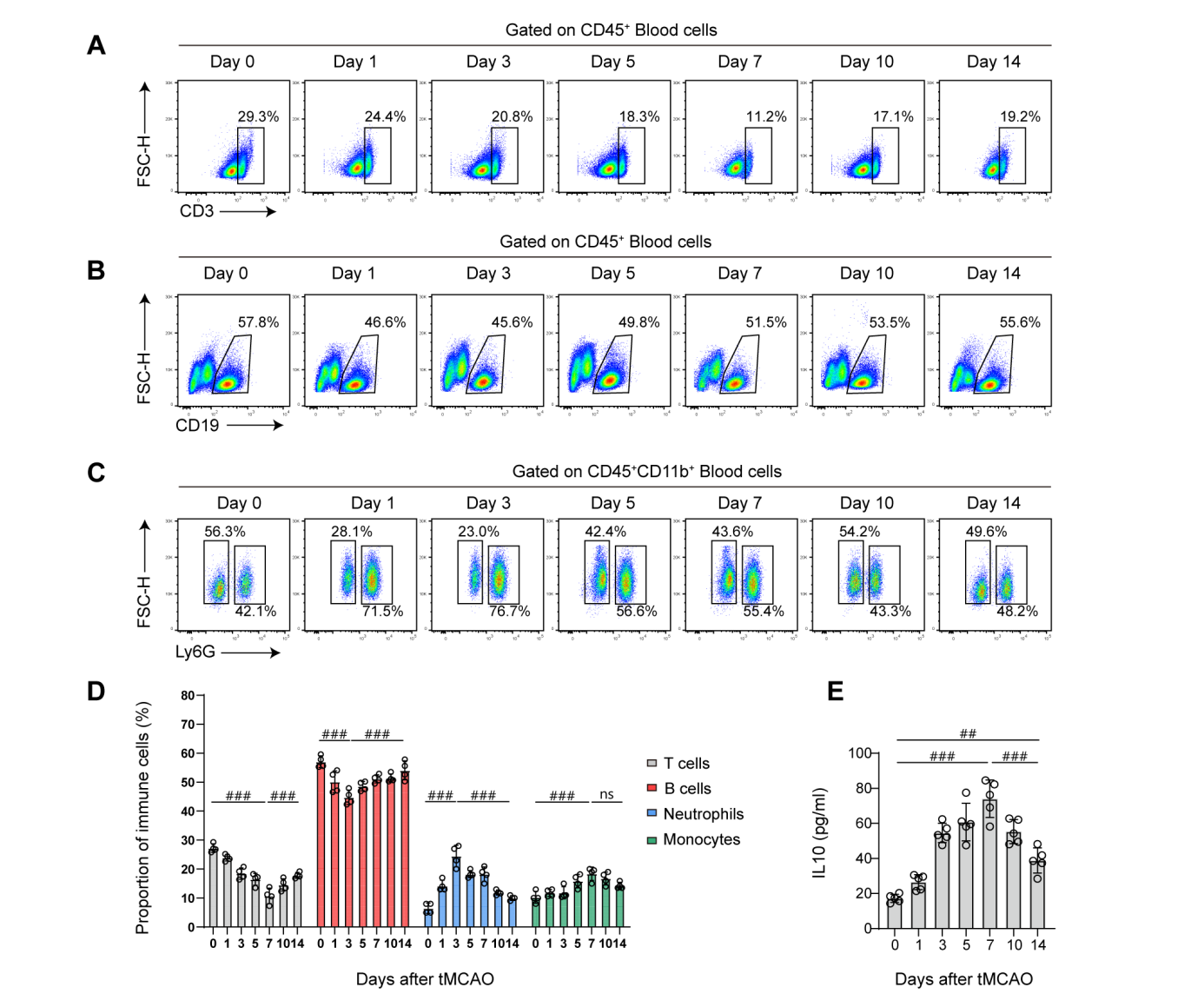


**Figure S4 Dynamic changes in peripheral immune profiles during post-stroke immunosuppression.**

**(A-D)** Flow cytometry analysis of peripheral blood leukocyte subsets from day 0 to 14 post-stroke. Proportions of T cells (CD45⁺CD3⁺) (A) and B cells (CD45⁺CD19⁺) (**B**). (**C**)Proportions of neutrophils (CD45⁺CD11b⁺Ly6G⁺) and monocytes (CD45⁺CD11b⁺Ly6G^-^). (**D**) Composite quantification of leukocyte kinetics. (**E**) Serum IL-10 levels measured by ELISA. Data represent mean ± SD; *n* = 4 per group. FDR-corrected *p*-values (*q-values*) were calculated, ###*q* < 0.001, by one-way ANOVA (mean ± SD).


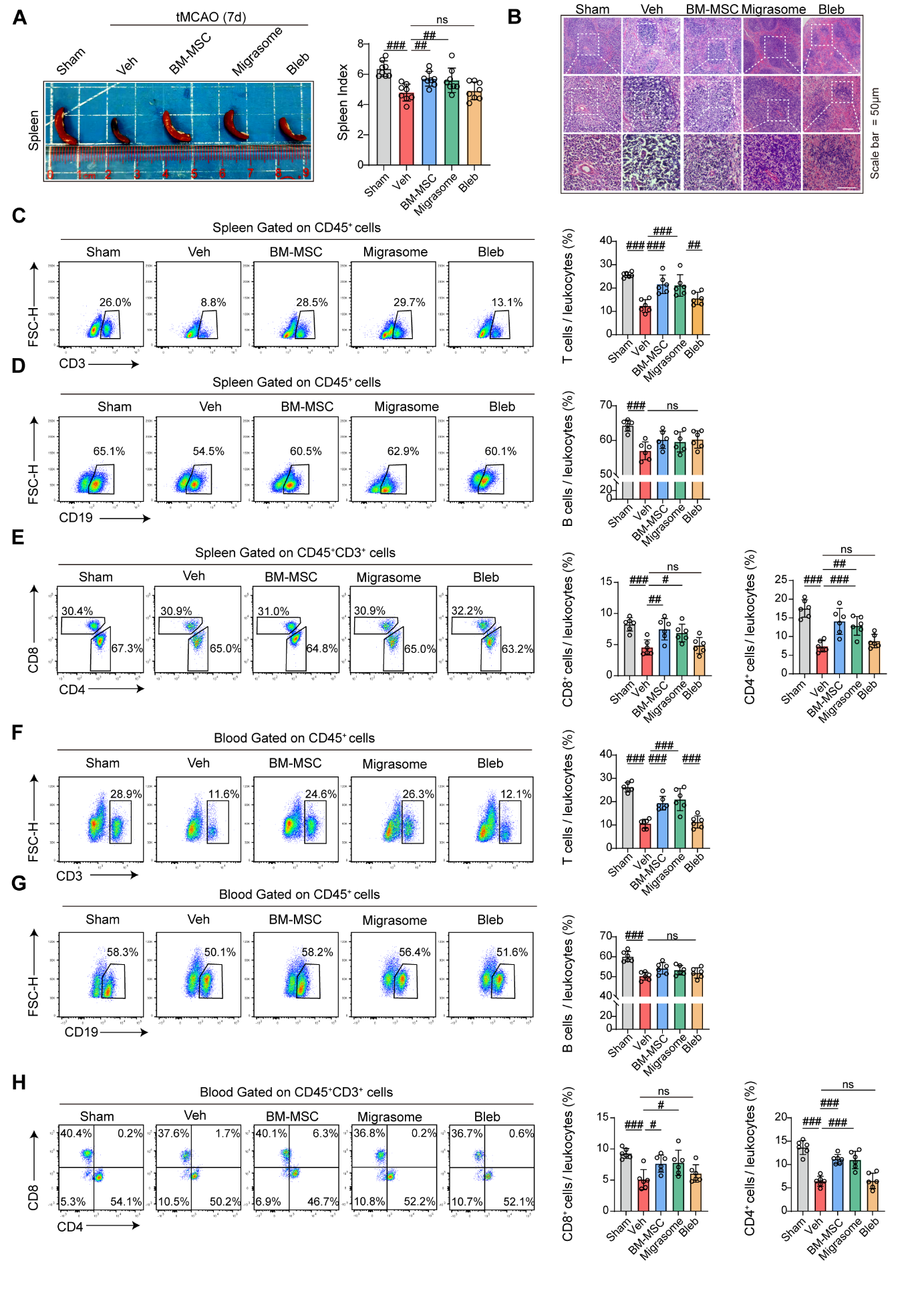


**Figure S5. Peripheral Immune Evaluation in Young Mice.**

Male wild-type C57/Bl6 mice underwent 60-minute tMCAO followed by intravenous administration of BM-MSCs (2×10⁶ cells/mouse), migrasomes (10 mg/kg/mouse, isolated from TSPAN4-GFP-overexpressing BM-MSCs), Bleb-pretreated BM-MSCs (2×10⁶ cells/mouse), or vehicle (Veh) 2 hours post-reperfusion. **(A)** Left: Gross spleen images at 7 days post-tMCAO. Right: Spleen index (spleen wet weight (mg) / mouse body weight (g) quantification (*n*=8 per group). FDR-corrected *p*-values (*q-values)* were calculated, ##*q* < 0.01, ###*q* < 0.001, one-way ANOVA (mean ± SD). **(B)** Representative H&E-stained spleen sections (*n*=3 per group; experiments repeated three times). **(C-H)** Flow cytometry analysis of splenic and peripheral blood immune cells, and the corresponding statistical analysis were displayed (*n*=6 per group). **(C-D, F-G)** Proportions of T cells (CD45^+^CD3^+^) and B cells (CD45^+^CD19^+^). **(E, H)** Proportions of CD4^+^ (CD45^+^CD3^+^CD4^+^) and CD8^+^ (CD45^+^CD3^+^CD8^+^) T cells. FDR-corrected *p*-values (*q-values)* were calculated, #*q* < 0.05, ##*q* < 0.01, ###*q* < 0.001, one-way ANOVA (mean ± SD).


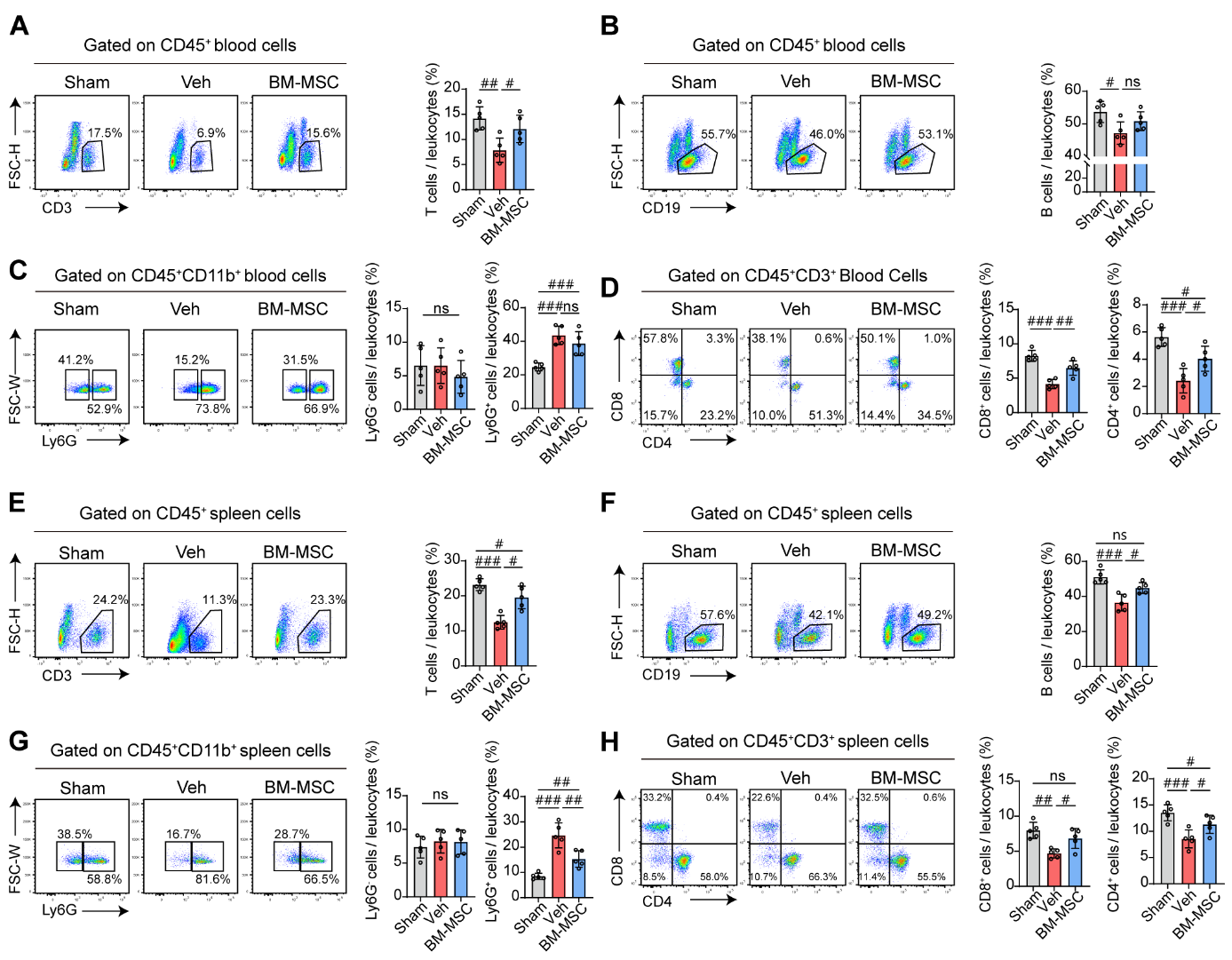


**Figure S6. Peripheral Immune Evaluation in Aged Mice.**

18 months male wild-type C57/Bl6 mice underwent 60-minute tMCAO followed by intravenous administration of BM-MSC (2×10⁶ cells/mouse), or vehicle (Veh) 2 hours post-reperfusion. (**A-D**) Flow cytometry analysis of peripheral blood immune cells, and the corresponding statistical analysis were displayed (*n* = 5 per group). Proportions of blood T cells (CD45^+^CD3^+^) (**A**) and B cells (CD45^+^CD19^+^) (**B**). (**C**) Proportions of blood monocytes (CD45^+^CD11b^+^Ly6G^−^) and neutrophils (CD45^+^CD11b^+^Ly6G^+^). (**D**) Proportions of blood CD4^+^ (CD45^+^CD3^+^CD4^+^) and CD8^+^ (CD45^+^CD3^+^CD8^+^) T cells. (**E-H**) Flow cytometry analysis of spleen immune cells, and the corresponding statistical analysis were displayed (*n*=5 per group). Proportions of spleen T cells (CD45^+^CD3^+^) (**E**) and B cells (CD45^+^CD19^+^) (**F**). (**G**) Proportions of spleen monocytes (CD45^+^CD11b^+^Ly6G^−^) and neutrophils (CD45^+^CD11b^+^Ly6G^+^). (**H**) Proportions of spleen CD4^+^ (CD45^+^CD3^+^CD4^+^) and CD8^+^ (CD45^+^CD3^+^CD8^+^) T cells. FDR-corrected *p*-values (*q-values*) were calculated, #*q*< 0.05, ##*q* < 0.01, ###*q* < 0.001, by one-way ANOVA (mean ± SD).


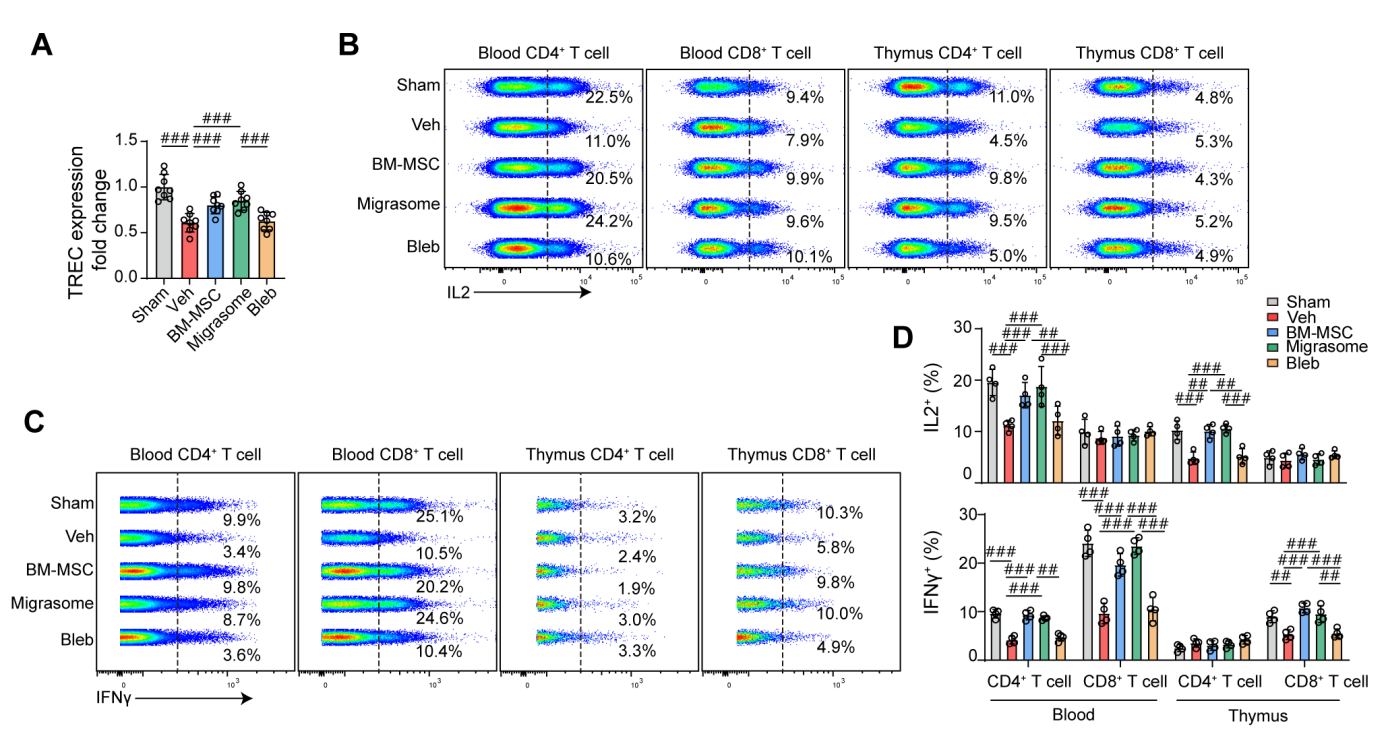


**Figure S7. Functional assessment of thymic output and T-cell competence.**

(**A**) Quantitative PCR analysis of TREC in peripheral blood, normalized to sham controls. (**B-C**) Flow cytometry analysis of intracellular cytokine production in peripheral blood and thymic T cells at day 7 post-stroke following 6-hour PMA/ionomycin stimulation: (**B**) Representative plots of IL-2⁺ cells. (**C**) Representative plots of IFNγ⁺ cells. (**D**) Quantification of IL-2⁺ and IFNγ⁺ T-cell subsets. Data represent mean ± SD; *n* = 4 per group. FDR-corrected *p*-values (*q-values)* were calculated, ##*q* < 0.01, ###*q* < 0.001, by one-way *ANOVA* (mean ± SD).


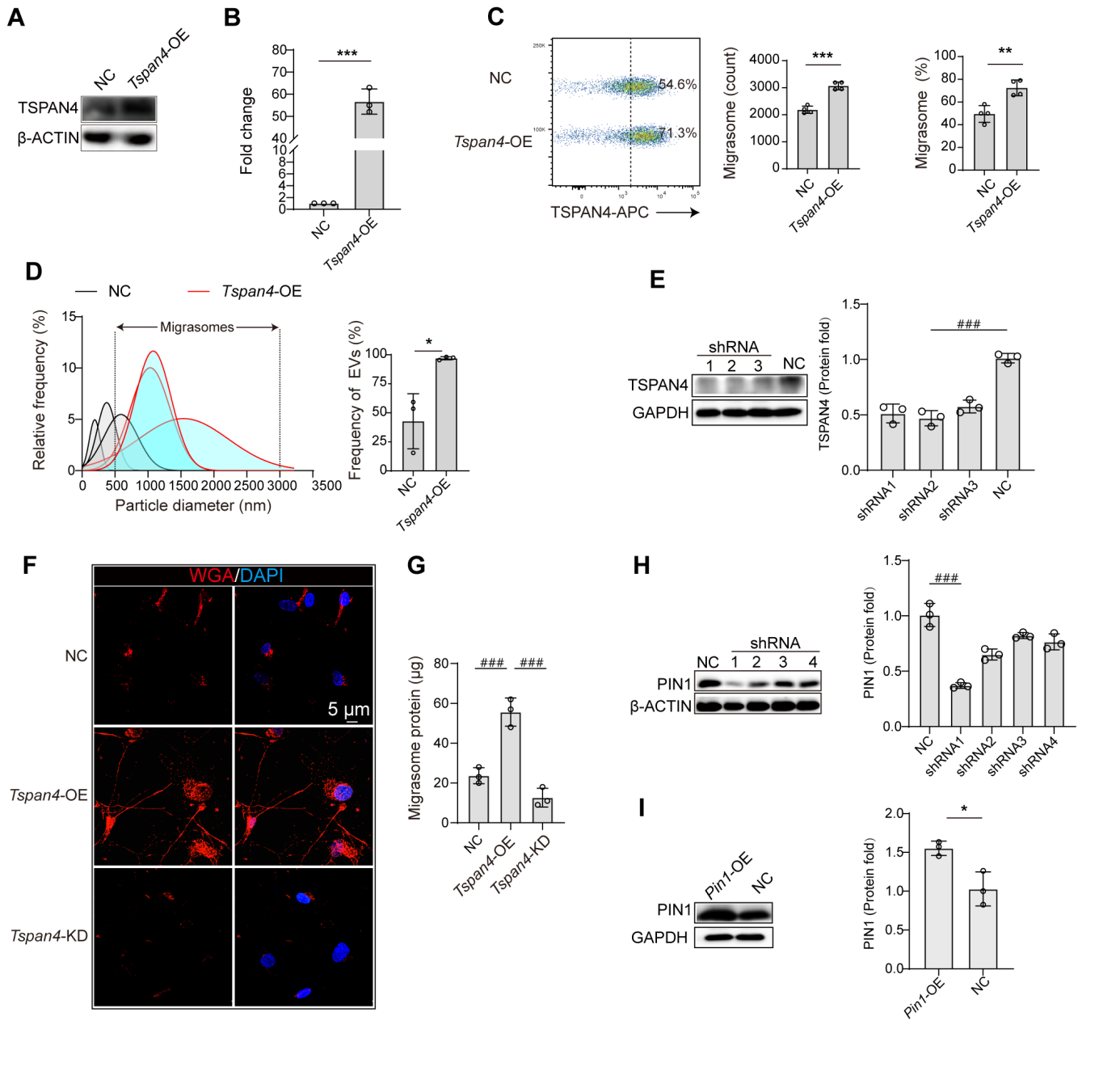


**Figure S8. Validation of engineered BM-MSC models for migrasome research.**

(**A-D**) TSPAN4 was overexpressed in BM-MSC via lentiviral infection to enhance migrasome production. **(A)** Western blot (WB) analysis of TSPAN4 protein expression in BM-MSC post-lentiviral infection. Representative images shown (experiments repeated three times). **(B)** RT-PCR analysis of TSPAN4 mRNA levels in BM-MSCs (*n*=3 per group, ****p* < 0.001, by Student' s *t*-test (mean ± SD). **(C)** Nanoparticle flow cytometry quantification of TSPAN4-positive migrasome particles (*n*=3 per group). Bar graphs show statistical results. ***p* < 0.01, ****p* < 0.001, by Student' s *t*-test (mean ± SD). **(D)** Diameter distribution of migrasome particles in *Tspan4*-OE versus control groups. **p* < 0.05, by Student' s *t*-test (mean ± SD). (**E**) *Tspan4* knockdown in BM-MSC via shRNA interference. Western blot of TSPAN4 expression in BM-MSC with quantification. Data represent mean ± SD; *n* = 3 per group. FDR-corrected *p*-values (*q-values*) were calculated, ###*q* < 0.001, by one-way ANOVA (mean ± SD). (**F**) Representative images of BM-MSC stained with wheat germ agglutinin (WGA, red) to label migrasomes. (**G**) Quantification of migrasome production by measuring the total protein content isolated from equal numbers (1×10⁶) of control (NC), *Tspan4*^OE^, and *Tspan4*^KD^ BM-MSC (*n*=3 independent isolations). FDR-corrected *p*-values (*q-values*) were calculated, ###*q* < 0.001, by one-way ANOVA (mean ± SD). (**H**) *Pin1* knockdown in BM-MSC via shRNA interference. Western blot of PIN1 expression in BM-MSC with quantification. Data represent mean ± SD; *n* = 3 per group. FDR-corrected *p*-values (*q-values*) were calculated, ###*q* < 0.001, by one-way ANOVA (mean ± SD). (**I**) Western blot analysis of PIN1 protein overexpression in BM-MSC post-lentiviral infection. Representative images shown (experiments repeated three times).


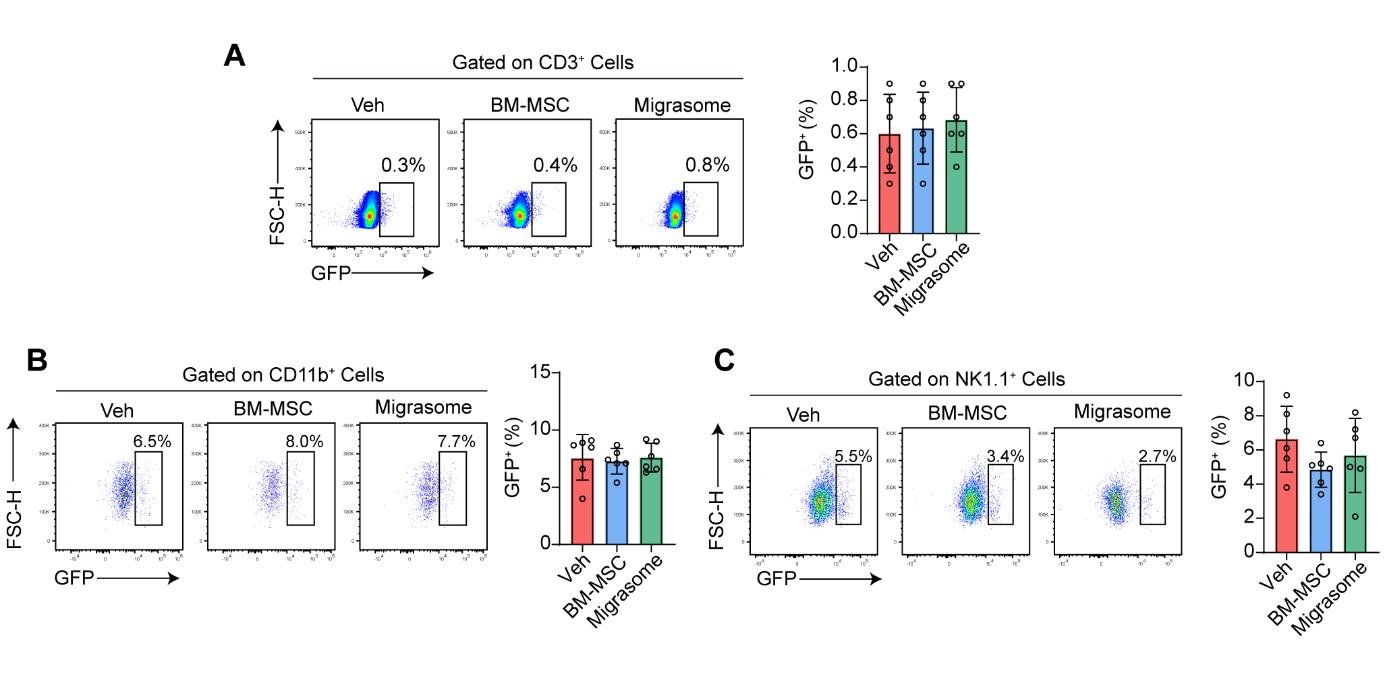


**Figure S9. Flow Cytometric Tracking of GFP Signal in Thymus of tMCAO Mice.**

Male wild-type C57/Bl6 mice underwent 60-minute tMCAO followed by intravenous administration of GFP-labeled BM-MSCs (2×10⁶ cells/mouse), migrasomes (10 mg/kg/mouse, isolated from TSPAN4-GFP-overexpressing BM-MSC), or vehicle (Veh) 2 hours post-reperfusion. Thymic tissues were harvested for flow cytometry analysis to track GFP signal. **(A)** Proportions of GFP^+^ cells in T cells (CD3^+^). **(B)** Proportions of GFP^+^ cells in myeloid cells (CD11b^+^) and (**C**) NK cells (NK1.1^+^). There was no significant difference among all groups (mean ± SD; *n*=6 per group).


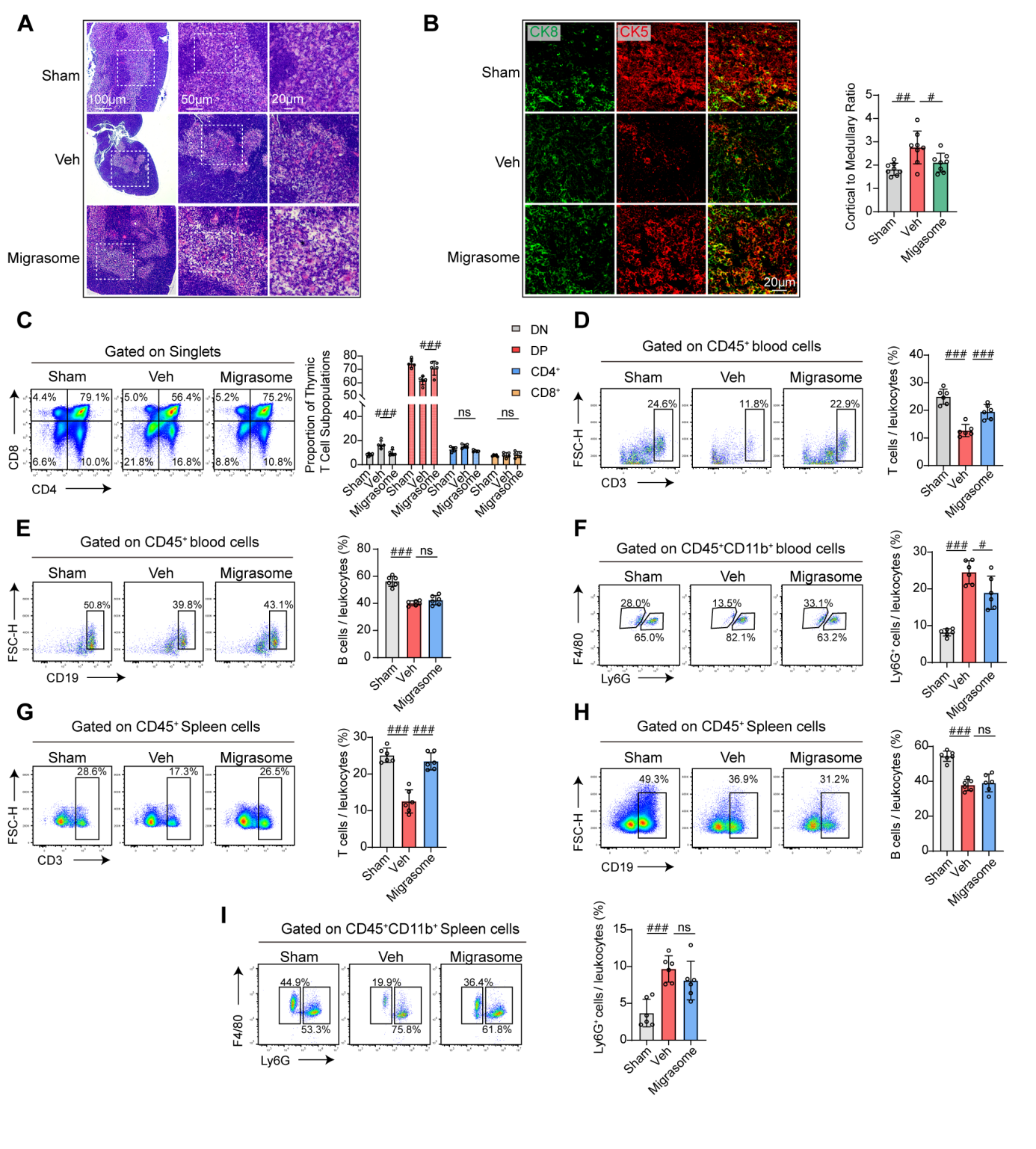


**Figure S10. BM-MSC-derived migrasomes promote thymic regeneration and systemic immune restoration in female mice after ischemic stroke.**

Female wild-type C57Bl/6 mice (8-week-old) were subjected to tMCAO followed by reperfusion. Mice were intravenously administered with either BM-MSC-derived migrasomes (10 mg/kg) or vehicle (Veh) as a single dose at 2 hours post-reperfusion. (**A**) Representative H&E-stained thymic sections (*n*=3 per group). (**B**) Immunofluorescence staining of the thymus: CK5 (red, medulla) and CK8 (green, cortex). The bar graphs show the cortical-to-medullary area ratios (*n*=8 per group). FDR-corrected *p*-values (*q*-values) were calculated, #*q* < 0.05, ##*q* < 0.01, one-way ANOVA (mean ± SD). (**C**) Thymic T-cell subset profiling: proportions of double-negative (DN, CD4^−^CD8^−^), double-positive (DP, CD4^+^CD8^+^), CD4^+^, and CD8^+^ T cells (*n*=8 per group). FDR-corrected *p*-values (*q*-values) were calculated, ###*q* < 0.001, one-way ANOVA (mean ± SD). (**D-F**) Flow cytometry analysis of peripheral blood immune cells, and the corresponding statistical analysis were displayed (*n* = 6 per group). Proportions of blood T cells (CD45^+^CD3^+^) (**D**) and B cells (CD45^+^CD19^+^) (**E**). (**F**) Proportions of blood monocytes (CD45^+^CD11b^+^F480^+^) and neutrophils (CD45^+^CD11b^+^Ly6G^+^). (**G-I**) Flow cytometry analysis of spleen immune cells, and the corresponding statistical analysis were displayed (*n*=6 per group). Proportions of spleen T cells (CD45^+^CD3^+^) (**G**) and B cells (CD45^+^CD19^+^) (**H**). (**I**) Proportions of spleen monocytes (CD45^+^CD11b^+^F480^+^) and neutrophils (CD45^+^CD11b^+^Ly6G^+^). FDR-corrected *p*-values (*q-values*) were calculated, #*q*< 0.05, ###*q* < 0.001, by one-way ANOVA (mean ± SD).


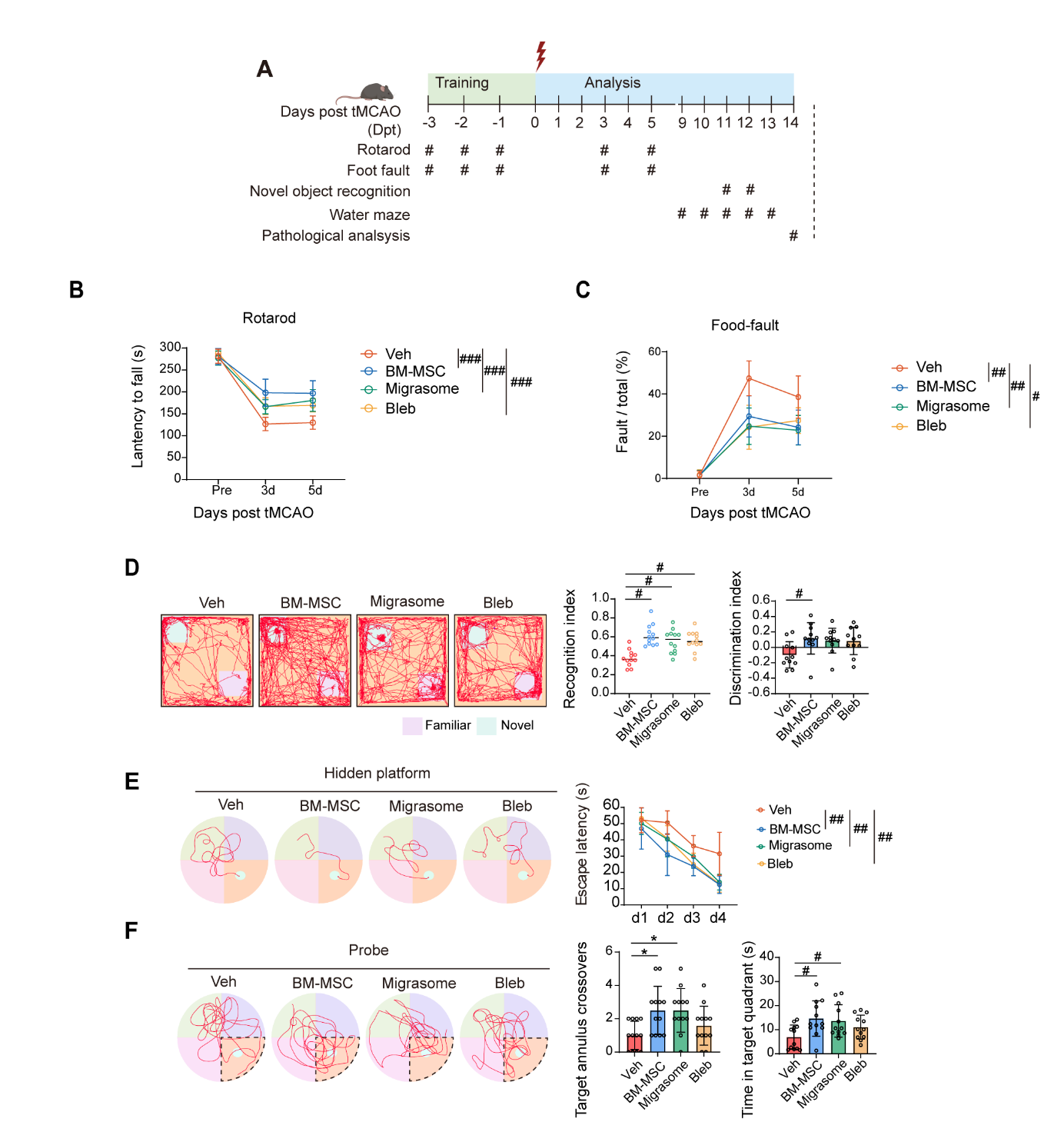


**Figure S11. Behavioral Assessments in tMCAO Mice.**

Male wild-type C57/Bl6 mice underwent 60-minute tMCAO followed by intravenous administration of BM-MSCs (2×10⁶ cells/mouse), migrasomes (10 mg/kg, isolated from TSPAN4-GFP-overexpressing BM-MSCs), blebbistatin (Bleb)-pretreated BM-MSCs (2×10⁶ cells/mouse), or vehicle (Veh) 2 hours post-reperfusion. **(A)** Schematic timeline of behavioral tests. (B-C) Motor-sensory function evaluation: **(B)** Rotarod latency (*n*=12 per group). **(C)** Foot-fault rate (*n*=12 per group). FDR-corrected *p*-values (*q-values)* were calculated, #*q* < 0.05, ##*q* < 0.01, ###*q* < 0.001 (BM-MSC, Migrasome, or Bleb vs. Veh at day 5), two-way ANOVA (mean ± SD). **(D)** Novel object recognition test (training and testing at day 11 post-tMCAO): recognition index (RI = Tnovel / [Tnovel+Tfamiliar] ×100%), and discrimination index (DI = [Tnovel-Tfamiliar] / [Tnovel+Tfamiliar]) (*n*=12 per group). FDR-corrected *p*-values (*q-values)* were calculated, #*q* < 0.05, one-way ANOVA (mean ± SD). **(E-F)** Morris water maze: **(E)** Escape latency during hidden platform trials (days 9-12 post-tMCAO, one trial/day; *n*=12 per group). FDR-corrected *p*-values (*q-values)* were calculated, ##*q* < 0.01, (BM-MSC and migrasome vs. Veh at day 4), by two-way ANOVA (mean ± SD). **(F)** Probe trial (day 13): Target annulus crossovers (left, by Wilcoxon rank-sum test, **p* < 0.05) and time in target quadrant (right, FDR-corrected *p*-values (*q-values)* were calculated, #*q* < 0.05, by one-way ANOVA; mean ± SD).


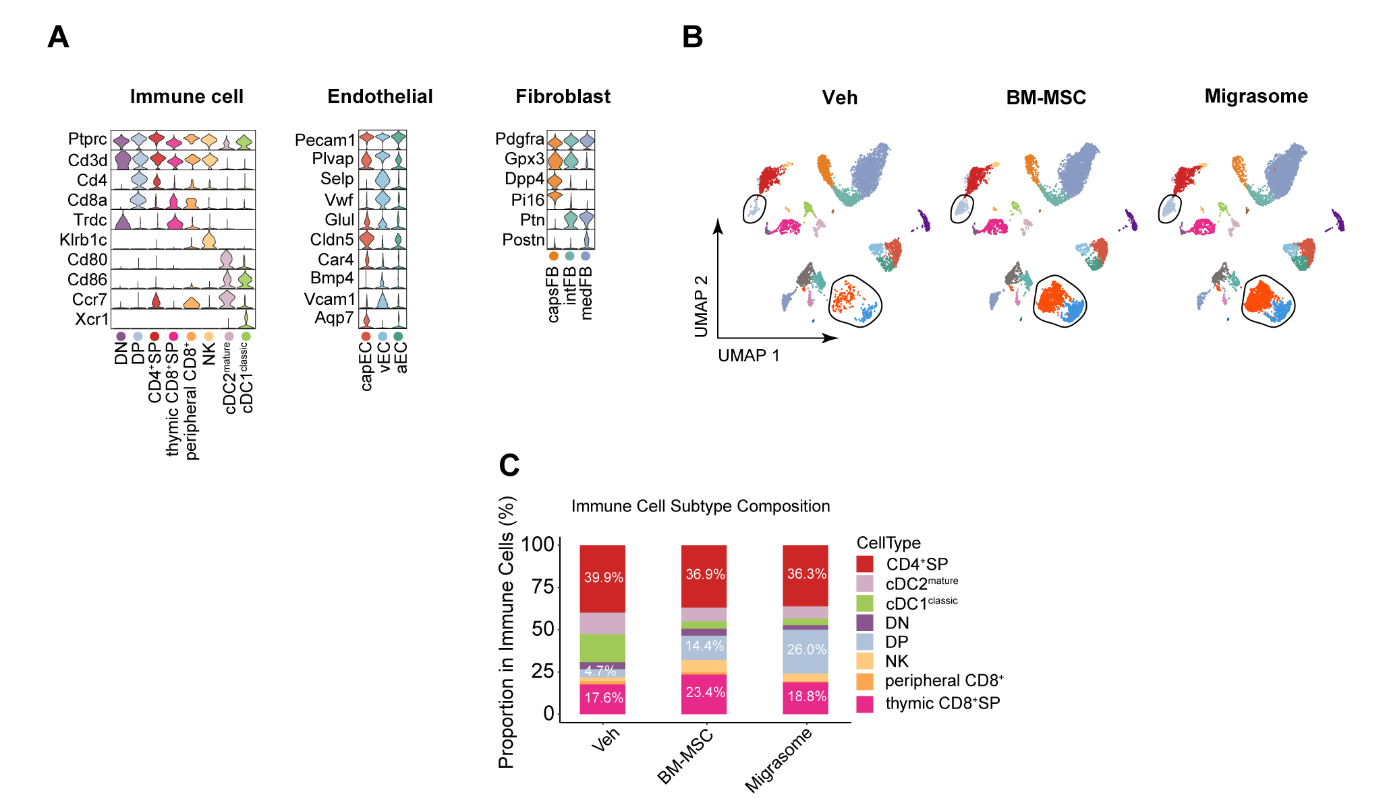


**Figure S12. Comprehensive Analysis of Non-epithelial thymic Populations in scRNA-seq Data.**

(**A**) Violin plots demonstrating expression distributions of lineage markers. (**B**) Integrated UMAP visualization stratified by treatment: Veh, BM-MSC, Migrasome. Solid lines contours highlight clusters with significant inter-group compositional differences. (**C**) Stacked bar plot quantifying proportional changes in immune cell subsets defined by established markers.


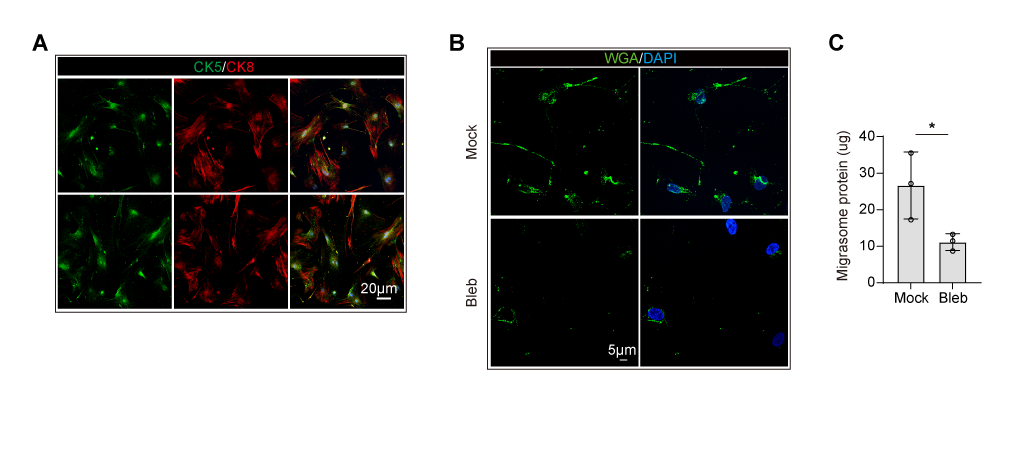


**Figure S13. In Vitro Isolation of TECs and Evaluation of Migrasomes from Bleb-Treated BM-MSCs.**

Thymic epithelial cells (TECs) were isolated and cultured from postnatal day 6 neonatal mice. **(A)** Immunofluorescence staining of TEC: CK5 (red, TEC marker), CK8 (green, TEC marker). Experiments repeated three times; representative images shown. **(B)** WGA staining (green, migrasome marker) showing reduced migrasome production in Bleb-pretreated BM-MSC. Experiments repeated three times; representative images shown. **(C)** Protein content of migrasomes isolated from equal numbers of BM-MSC (1×10⁶ cells) with or without Bleb treatment, quantified by BCA assay (*n*=3 per group). **p* < 0.05, by Student' s *t*-test (mean ± SD).


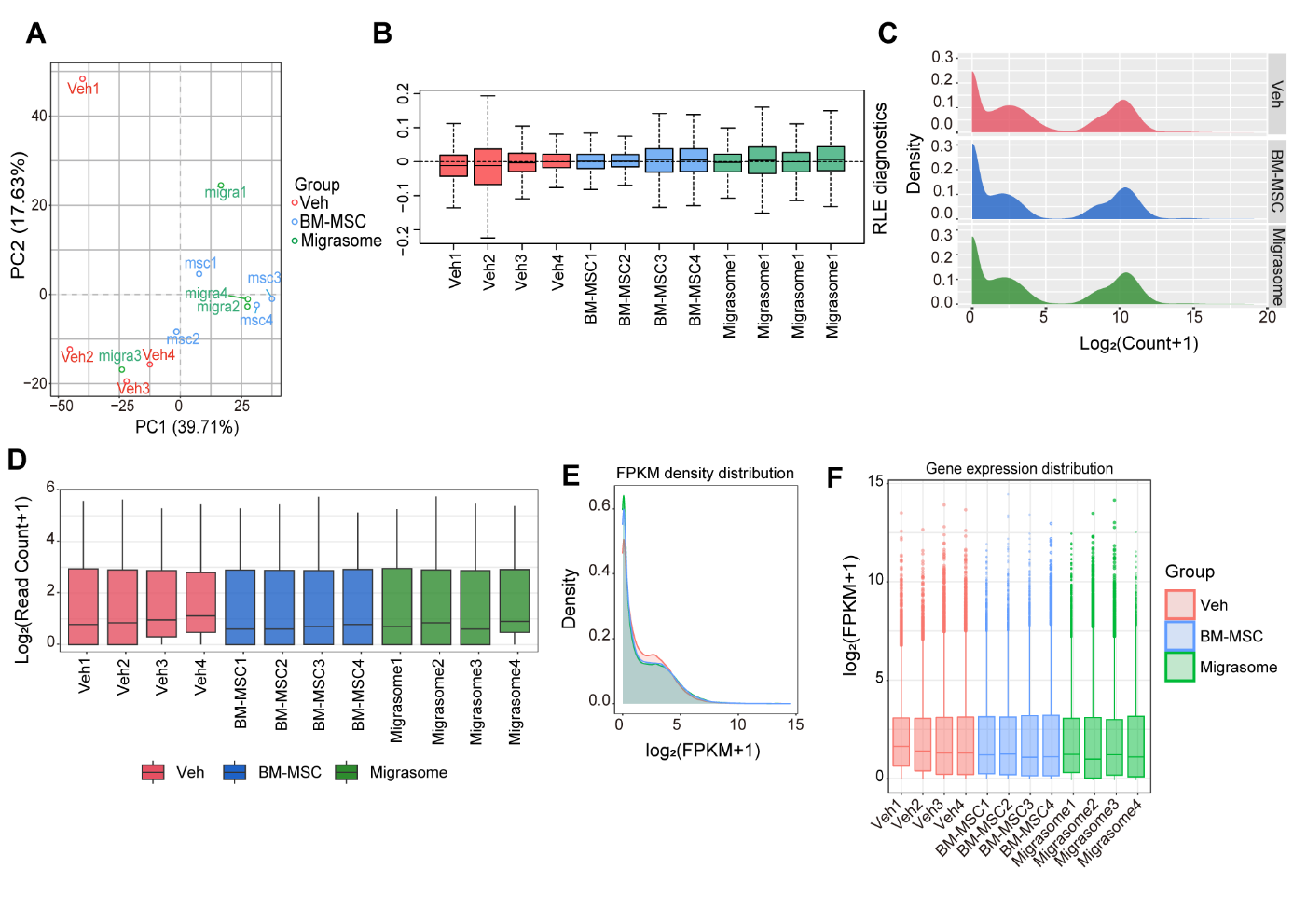


**Figure S14. Quality Control of bulk RNA-seq Data.**

Male wild-type C57BL/6 mice underwent 60-minute transient middle cerebral artery occlusion (tMCAO) followed by reperfusion. BM-MSC (2×10⁶ cells/mouse), migrasomes (10 mg/kg), or vehicle (Veh) were administered intravenously 2 hours post-reperfusion. Thymic tissues were harvested at 7 days post tMCAO for RNA sequencing (*n* = 4 per group). (**A**) Principal component analysis (PCA) based on FPKM demonstrates clear separation between treatment groups (BM-MSC, Migrasome) and the Veh group. (**B**) Relative Log Expression (RLE) diagnostics: Distribution of log₂(CPM+1) values after normalization by edgeR. All samples exhibit medians centered near zero (IQR < 0.2), indicating controlled technical variation. (**C**) Density plots of read-count distributions: Overlapping curves of log₂(Count+1) transformed data across groups suggest minimal technical noise. (**D**) Boxplots of read-count distributions: Sample medians concentrated within log₂(Count+1) = 0–2 range. (**E**) Density plots of FPKM distributions: High curve coherence of log₂(FPKM+1) values across groups. (**F**) Boxplots of FPKM distributions: Sample medians within log₂(FPKM+1) = 0-5 range. Statistical analysis: PCA performed via linear algebra dimensionality reduction (R package stats); RLE and distribution analyses using edgeR and ggplot2; normalization details in Methods.


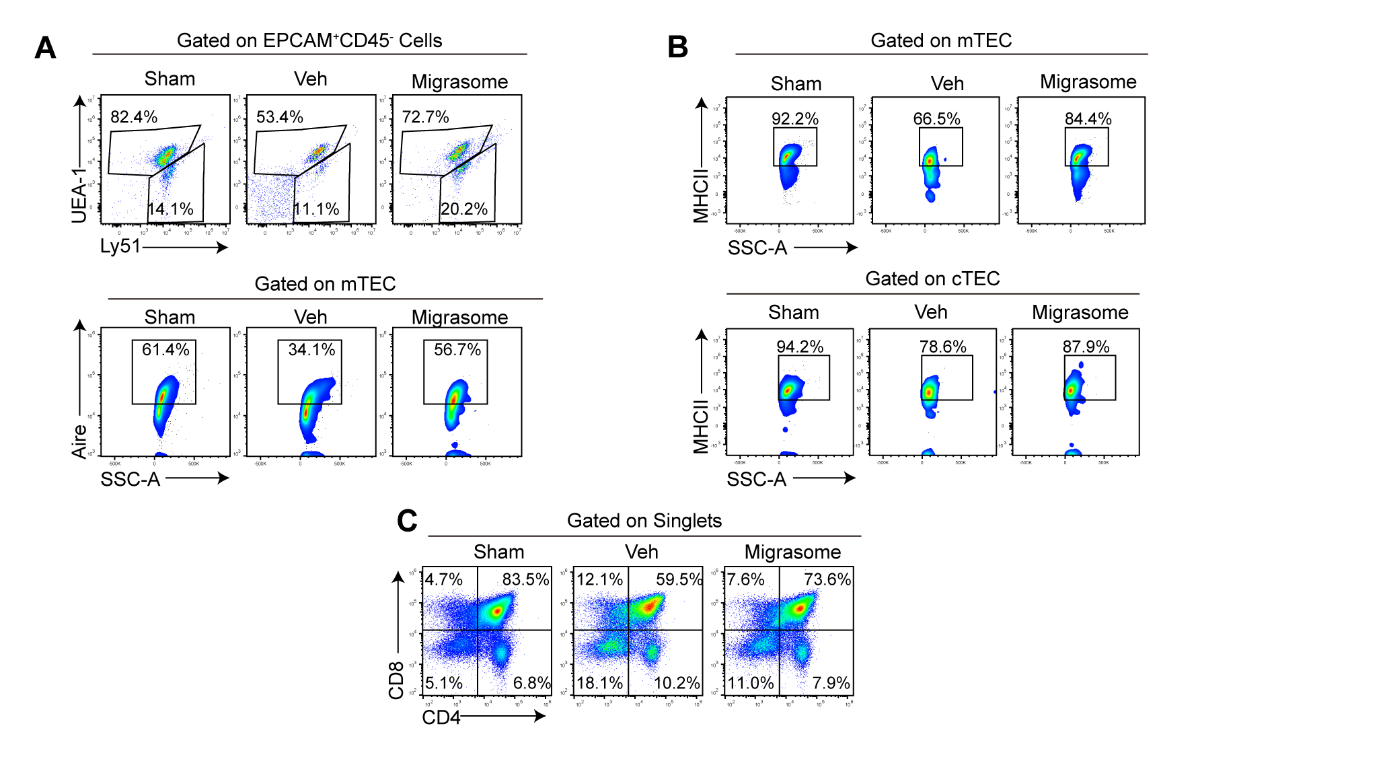


**Figure S15. Flow Cytometry Analysis of Thymic Epithelial Cells and T-Cell Subsets in Migrasome-Treated tMCAO Mice.**

Flow cytometry evaluation of the effects of migrasome monotherapy on thymic epithelial cells (TEC) and T-cell subsets in tMCAO mice. **(A)** Proportions of mTEC (EPCAM^+^CD45^-^UEA-1^+^Ly51^-^) and cTEC (EPCAM^+^CD45^-^UEA-1^-^Ly51^+^) in three groups (Sham, Veh, migrasome). **(B)** Proportions of MHC II^+^ and AIRE^+^ cells in mTEC and MHC II^+^ cells in cTEC. **(C)** Thymic T-cell subset profiling: DN, DP, CD4^+^, and CD8^+^ T cells. The statistical diagram is shown in Figure 3.

**Table S1. Primers used in the study.**

| **Gene** | **Sequence** |
| --- | --- |
| **Homo sapiens** | |
| TSPAN4 Forward | TGTCCGCTTGACTGACAGC |
| TSPAN4 Reverse | GCGCTTCAGTTCTGGGCT |
| GAPDH Forward | TCGGAGTCAACGGATTTGGT |
| GAPDH Reverse | TTCCCGTTCTCAGCCTTGAC |
| **Mus musculus** | |
| mTREC Forward | CCAAGCTGACGGCAGGTTT |
| mTREC Reverse | AGCATGGCAAGCAGCACC |
| mTREC Probe | FAM/TGCTGTGTG/XEN/CCCTGCCCTGCC/BHQ1 |
| mTRAC Forward | TGACTCCCAAATCAATGTG |
| mTRAC Reverse | GCAGGTGAAGCTTGTCTG |
| mTRAC Probe | FAM/TGCTGGACA/ZEN/TGAAAGCTATGGA/BHQ1 |
